# Recent advances in the study of zika virus structure, drug targets, and inhibitors

**DOI:** 10.3389/fphar.2024.1418516

**Published:** 2024-07-01

**Authors:** Yingqi Feng

**Affiliations:** Beijing Key Laboratory for Green Catalysis and Separation and Department of Chemical Engineering, College of Materials Science & Engineering, Beijing University of Technology, Beijing, China

**Keywords:** ZIKV, drug target, RdRp, MTase, inhibitor

## Abstract

Zika Virus (ZIKV) is a positive-strand RNA virus that can lead to Guillain-Barré syndrome or encephalitis in some individuals and hence presents a serious public health risk. Since the first outbreak of ZIKV in Brazil in 2015, no effective clinical inhibitors have been developed, making the development of effective ZIKV drugs an urgent issue that needs to be addressed. ZIKV belongs to the Flaviviridae family, and its structure includes three structural proteins, namely, capsular (C), premembrane (prM), and envelope (E) proteins, as well as seven nonstructural proteins, namely, NS1, NS2A, NS2B, NS3, NS4A, NS4B, and NS5. To provide a reference for the development of future ZIKV drugs, this paper reviews the structure of the ZIKV based on recent literature reports, analyzes the potential therapeutic targets of various proteins, and proposes feasible drug design strategies. Additionally, this paper reviews and classifies the latest research progress on several protease inhibitors, such as E protein inhibitors, NS2B-NS3 inhibitors, and NS5 inhibitors, so that researchers can quickly understand the current status of development and the interconnections among these inhibitors.

## 1 Introduction

ZIKV belongs to the family of Flaviviruses and can infect humans. Among those infected with ZIKV, about 20% will display clinical symptoms. Possible symptoms include Guillain-Barré Syndrome, thrombocytopenia, ocular damage, multi-organ failure, and testicular damage. Furthermore, among newborns born to pregnant women infected with ZIKV, approximately 80% of these infants will show signs of microcephaly (Plourde and Bloch, 2016). Besides mosquito-borne transmission, ZIKV may also be transmitted from mother to child and through sexual contact. In 2015, an outbreak of ZIKV occurred in Brazil, and by 2016, the epidemic had spread throughout the Americas. By the end of 2016, the Americas had reported over 5,00,000 cases of ZIKV infection (Crisanto-Lopez et al., 2023). In 2021, there was a minor outbreak of ZIKV in Kanpur, Uttar Pradesh, India. ZIKV poses a serious threat to public health, and the World Health Organization (WHO) has declared the ZIKV infection a public health emergency of international concern. Unfortunately, to date, there are no effective treatment drugs or vaccines against ZIKV on the market (Gorshkov et al., 2019).

**FIGURE 1:**
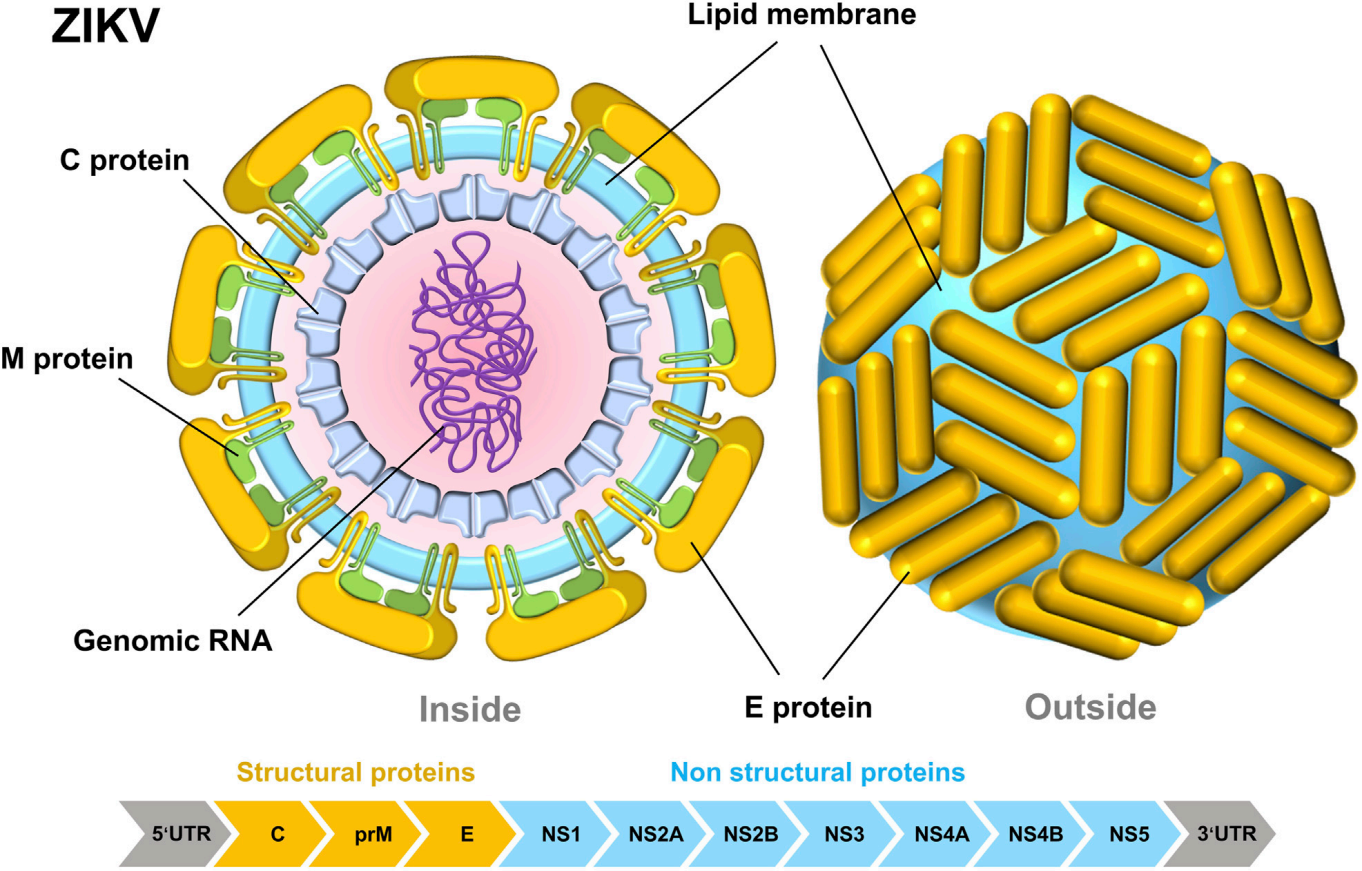
Internal structural diagram of ZIKV (left) and surface structure diagram (right). The schematic pieces were provided by Smart Medical Art and adapted from (http://www.servier.com). Servier Medical Art by Servier is licensed under a Creative Commons Attribution 3.0 Unported License.

ZIKV is a single-stranded, enveloped RNA virus. It includes around 10,794 nucleotides and is housed within a capsid. The ZIKV genome consists of two non-coding regions (NCR), the 5’and 3’NCR, and an open reading frame (ORF) that encodes multiple proteins. ZIKV’s compact dimensions enable it to enter host cells by receptor-mediated endocytosis (Mirza et al., 2022). ZIKV consists of three structural proteins: C, prM, and E proteins; and seven nonstructural proteins: NS1, NS2A, NS2B, NS3, NS4A, NS4B, and NS5 (Figure 1) (Sirohi and Kuhn, 2017). Since 2016, the crystal structures of all ZIKV protein fractions have been documented, with the exception of several nonstructural proteins (NS2A, NS4A, and NS4B). ZIKV’s general structure closely resembles those of other flaviviruses, such as Dengue Virus (DENV), West Nile Virus (WNV), Yellow Fever Virus (YFV), and Japanese Encephalitis Virus (JEV) (van den Elsen et al., 2023). ZIKV’s thick surface contributes to its thermal stability, enabling it to spread efficiently in hot climates (Lin et al., 2018).

Currently, no anti-ZIKV drugs have been approved for commercial use, increasing the urgency for ZIKV research. Over the past few years, while several active compounds have emerged, there is still a considerable distance to go before we can effectively control the progression of ZIKV. Up to the present day, the understanding of ZIKV is still deficient, particularly in the deep research into the mechanisms of action of drugs. Some reports have reviewed drugs targeting NS2B-NS3 and NS5 in ZIKV (Voss and Nitsche, 2020; Nascimento et al., 2021; Delgado-Maldonado et al., 2023), but these reports mostly focus on individual targets and do not provide a systematic and comprehensive analysis of ZIKV targets from the perspective of protein structure (Bernatchez et al., 2020). Furthermore, these studies do not analyze the characteristics and interrelationships of various inhibitors. This paper comprehensively summarizes for the first time the drug action targets on the various proteins of ZIKV, some of which have been confirmed, while others remain hypothetical. Such systematic work is helpful for drug researchers to gain an in-depth and comprehensive understanding of ZIKV, greatly aiding the development of drugs against the virus. Furthermore, this paper describes the infection mechanism of ZIKV from a structural level and summarizes the reported anti-ZIKV compounds. These compounds are subdivided into several major structural categories, which facilitate a comprehensive understanding of the therapeutic drugs for ZIKV.

## 2 Life cycle of ZIKV

**FIGURE 2:**
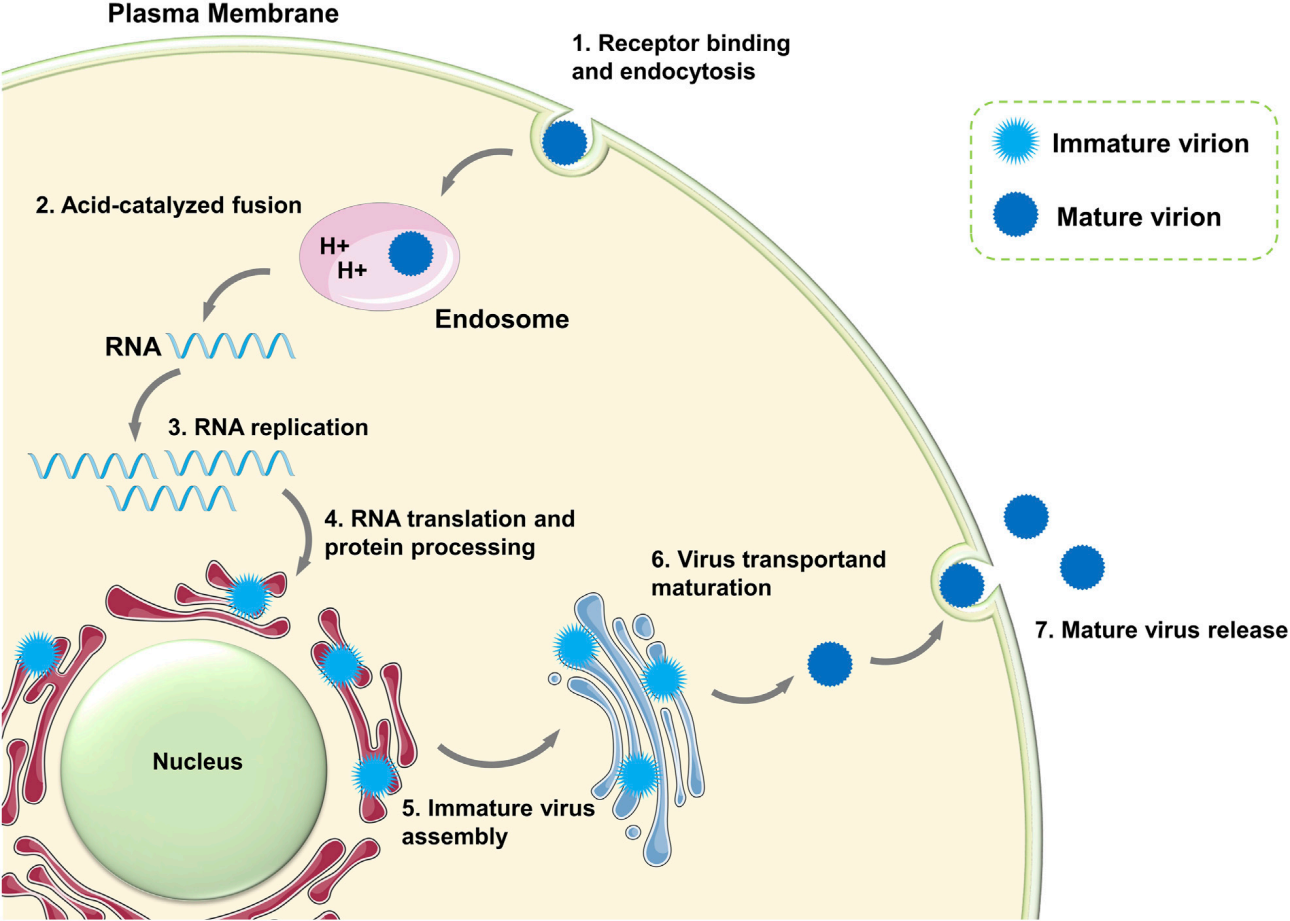
Life cycle of ZIKV. The schematic pieces were provided by Smart Medical Art and adapted from (http://www.servier.com). Servier Medical Art by Servier is licensed under a Creative Commons Attribution 3.0 Unported License.

In the life cycle of ZIKV (Figure 2), mature ZIKV initially identifies the host receptor through the E protein on its surface, specifically binds to it, and initiates endocytosis (Perera et al., 2008). Upon entering the cell, the E protein of ZIKV undergoes a conformational change from a dimer to a trimer because of the acidic environment of the host cytoplasm, leading to exposure of the fusion loop (FL) of the E protein (Alcon et al., 2002). The viral membrane fuses with the cell endosome membrane through FL, releasing the (+)-strand viral genomic RNA from the nucleocapsid into the cytoplasm (Bressanelli et al., 2004; Stiasny et al., 2007). Subsequently, viral RNA begins to translate viral polyprotein. The newly synthesized E and prM proteins encapsulate the RNA and C proteins and assemble into “sharp” immature viral particles (Saiz et al., 2016). The immature virions are carried to the Golgi and trans-Golgi network (TGN) of the host cell, where their prM proteins are split into pr peptides and M proteins (M-E heterodimers), creating “smooth” mature virions. Ultimately, the fully developed daughter virus is expelled by cytophagy to initiate the subsequent viral life cycle (Lindenbach and Rice, 2003). The proteins (E, prM, and C) in the immature viral particles are transformed into the mature form (E, M, and C) in this process. In the entire life cycle of ZIKV, there are mainly three states—the immature state, mature state, and fusion state—that correspond to the non-infectious state, infectious state, and host membrane-binding state, respectively.

## 3 Structure and drug targets of ZIKV

Mature ZIKV is composed of structural proteins, nonstructural proteins, and ribosomal RNA genome. The E, M, and C proteins comprise the structural proteins. The M-E heterodimer is anchored to the lipid membrane by a transmembrane helix and establishes a particular connection with the C protein in the lipid envelope. prM proteins, the precursors of M proteins, are exclusively found in immature viruses. prM/M proteins and E proteins undergo a large conformational change during the life cycle of ZIKV, with distinct conformations in the immature, mature, and fusion states of the virus. The nonstructural proteins (NS1, NS2A, NS2B, NS3, NS4A, NS4B, and NS5) and ribosomal RNA genes are enclosed by structural proteins within the virus. The ribosomal RNA genome is the primary genetic material that relies on non-structural proteins for RNA replication. This work, based on the reported protein crystal structures, provides a detailed structural description of ZIKV structural and non-structural proteins and clearly identifies the potential drug-binding sites on each protein (Perera et al., 2024).

### 3.1 Structural proteins

The E proteins, prM/M proteins, and C proteins are important components of the ZIKV structure. They safeguard the virus’s genetic material, identify host receptors, and support viral replication. Therefore, structural proteins are key focus areas for the development of ZIKV inhibitors.

#### 3.1.1 E protein

The E protein of ZIKV is a crucial membrane protein that is essential for viral structural building, viral attachment, and viral penetration into the host cell. The E protein mediates viral contact with the receptor by interacting with the receptor on the surface of the host cell, which is known as viral attachment. Once the virus binds to the receptor, the E protein helps merge the virus with the host cell and transfers the ZIKV genetic material into the host cell through a sequence of intricate structural alterations, ultimately finalizing the virus’s entry into the host cell (Hu et al., 2021). Proper folding of the E protein is essential for the synthesis of prM-E during viral generation.

**FIGURE 3:**
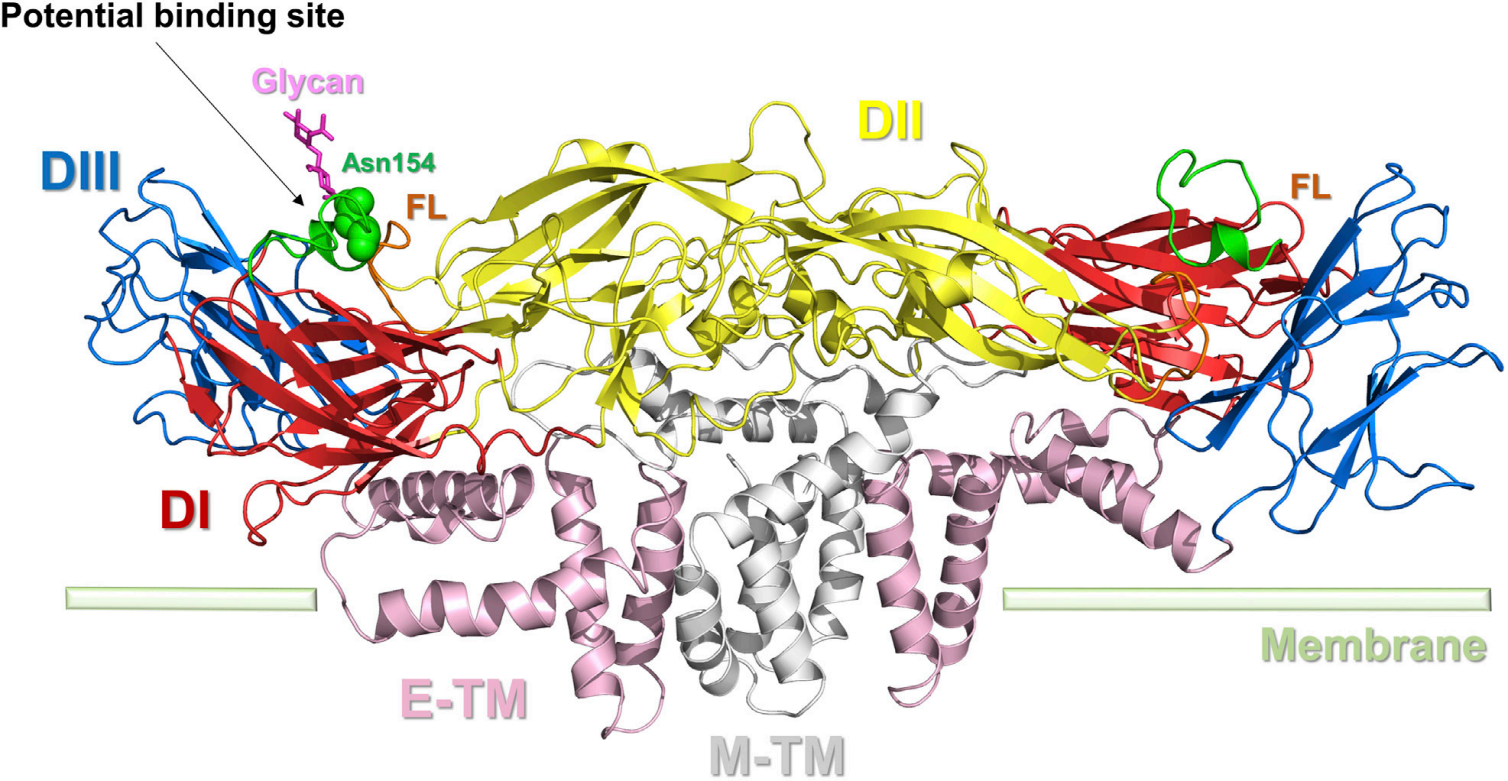
Dimer structure of the E protein of ZIKV. E protein domain I (DI): red; domain II (DII): yellow; domain III (DIII): blue; the transmembrane portion of the E protein (E-TM): pink; the transmembrane portion of the M protein (M-TM): gray; FL: brown. The key amino acid Asn154 is represented by a green sphere. Potential binding sites for inhibitors are indicated.

The E protein monomer (PDB ID: 5JHM) comprises three structural domains: a core β-barrel domain I (DI), an extended finger dimerization domain II (DII), and an immunoglobulin-like domain III (DIII) (Modis et al., 2004). The two individual E protein monomers can join together to create a dimer. The DI is structured as an eight-stranded β-barrel. Finger DII is composed of two segments and includes a fusion ring (FL) at the distal end (Figure 3). FL is crucial for the virus to merge with the host cell. DIII is autonomously folded and includes an antitrypsin receptor binding site. DI serves as the intermediary between DII and DIII. When virus-host cell fusion occurs, the DI-DII hinge structure undergoes a flipping motion in the acidic environment, wherein the FL in DII is released, and the DI-DIII hinge structure remains rigid. The E protein in ZIKV displays a distinct “herringbone” shape and contains a single glycosylation site at residue Asn154. Compared to other flaviviruses, the DI structure of ZIKV has a longer “150 loop” (residues 145–160), causing Asn154 to protrude beyond the surface. Notably, this 150-loop varies not only among ZIKV strains but also among other flavivirus members, which may impact the virus’s infectivity (Agrelli et al., 2019). DII in ZIKV is slightly nearer to the viral membrane than in DENV, and the radius of DIII is slightly longer in ZIKV than in DENV (Sirohi et al., 2016).

No specific pharmacological targets have been discovered for the E protein, similar to the C protein of ZIKV. Elfiky et al. carried out a computer-based simulation of a favorable binding between the ZIKV E protein and the receptor glucose-regulating protein 78 (GRP78) on the host cell membrane (Elfiky and Ibrahim, 2021). They determined that the optimal drug-binding site for the ZIKV E protein is located in the DIII structural domain. This study focuses on identifying therapeutic targets in the E protein and offers fresh insights into drug design research to create appropriate peptidomimetic inhibitors (Elfiky and Ibrahim, 2021). The DIII structural domain is situated near Asn154 and the FL section of the E protein and houses an antitrypsin receptor binding site; it is a promising focus for future E protein inhibitor research.

#### 3.1.2 prM/M proteins

**FIGURE 4:**
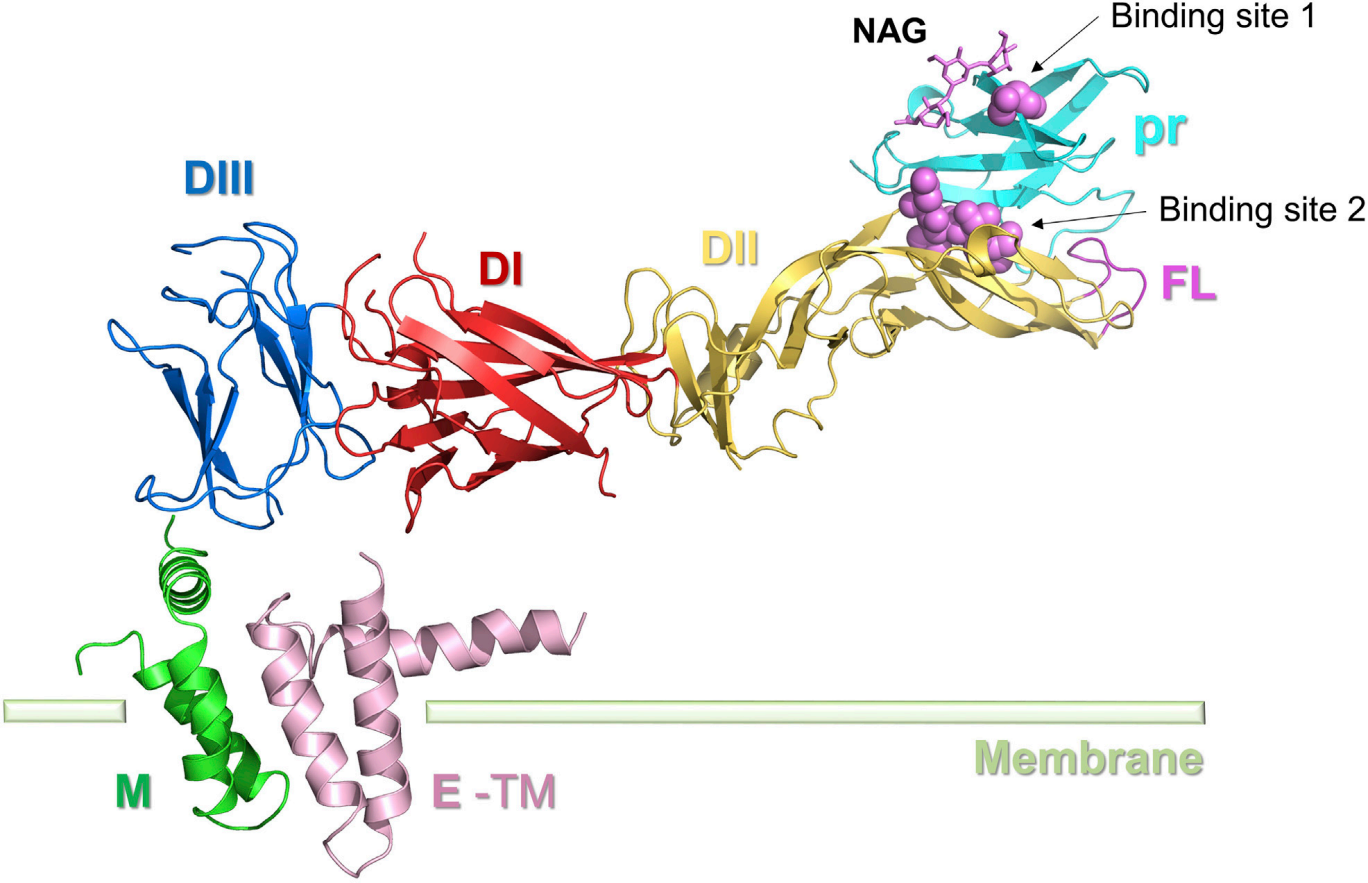
Structure of the ZIKV M protein. E protein domain I (DI): red; domain II (DII): yellow; domain III (DIII): blue; E-TM: pink. The pr protein (light blue) and M protein (green) are located at the two ends of the E protein, respectively. Inhibitor binding sites 1 and 2 are represented by spheres.

The prM protein is the precursor of the ZIKV M protein, also known as the pre-envelope protein, found in immature viruses (Figure 4). The prM protein is crucial for the virus’s assembly, release, maturation, and infection processes. During viral replication, the prM protein interacts with the E protein to create the precursor of the viral particle. After the formation of viral particles, the prM protein stabilizes the viral particle shape by interacting with the E protein. The prM protein structure shields the FL structure in E, crucial for receptor engagement, from irreversible conformational changes induced by the acidic Golgi environment, thus preventing its premature fusion with the host cell membrane. In addition to protecting the FL of E proteins during maturation, the prM protein also ensures the correct folding of E proteins (Lorenz et al., 2002). After ZIKV infection of the host cell, the prM protein is cleaved and altered to become the mature M protein. The M protein is a significant portion of the viral particle’s outer membrane and is involved in virus-host cell interactions and invasion processes (Dai et al., 2016; Mulgaonkar et al., 2020).

The prM protein comprises a pr peptide and an M protein. The pr structural domains are situated at the top of the prM protein spikes and cover the fusion loop (FL) of the E protein. In immature viruses, the prM-E complex assembles in the neutral environment of the endoplasmic reticulum to form trimeric spikes. When the virus is transported to the acidic environment of the exosomes, these spikes rearrange into dimer structures, covering the surface of the viral lipid envelope, thus forming the capsid proteins of the mature virus (Hu and Sun, 2019). The prM protein in mature viruses is cleaved in the TGN to form pr peptides and M proteins. The M protein comprises an N-terminal loop (M loop), a stem region, and two transmembrane helices. The M protein architectures of mature flaviviruses such as ZIKV, DENV2, and JEV viruses are all extremely conserved (Wang et al., 2017).

As prM plays a role in ensuring correct folding of the E protein, destabilizing the prM protein can affect the infectivity and pathogenicity of the virus by altering the character of the E protein and prM-E (Lorenz et al., 2002). This aspect shows great potential for drug development. N-acetyl-β-D-glucosidase (NAG) was identified as a natural ligand of prM (PDB ID: 5U4W) with a site near Thr4 (Prasad et al., 2017). Inhibitors can bind competitively to Thr4 sites, causing inhibition of prM, and the Thr4 glycosylation site is therefore considered as a possible small molecule binding site (Binding site 1). Most existing studies on the virtual design of prM protein inhibitors use NAG as the lead compound (Zheng et al., 2010; Mulgaonkar et al., 2020). Oliveira et al. identified five crucial residues (Gly102, His244, Thr70, Thr68, and Asn67) in the E protein (Binding site 2) that play a key role in maintaining the stability of the pr-E complex in the Golgi apparatus. This area could also be a promising drug target.

#### 3.1.3 C protein

The C protein of ZIKV is a structural protein that plays a role in the assembly and packaging of viral particles. It can interact with the RNA of the viral genome and assemble core particles within cells to encapsulate the viral genome. The mature C protein can also control virus replication or alter the host cell environment, and it interacts with various host proteins, including B23, Jab1, hnRNP K, and hnRNP A2 (Zhang et al., 2021).

**FIGURE 5:**
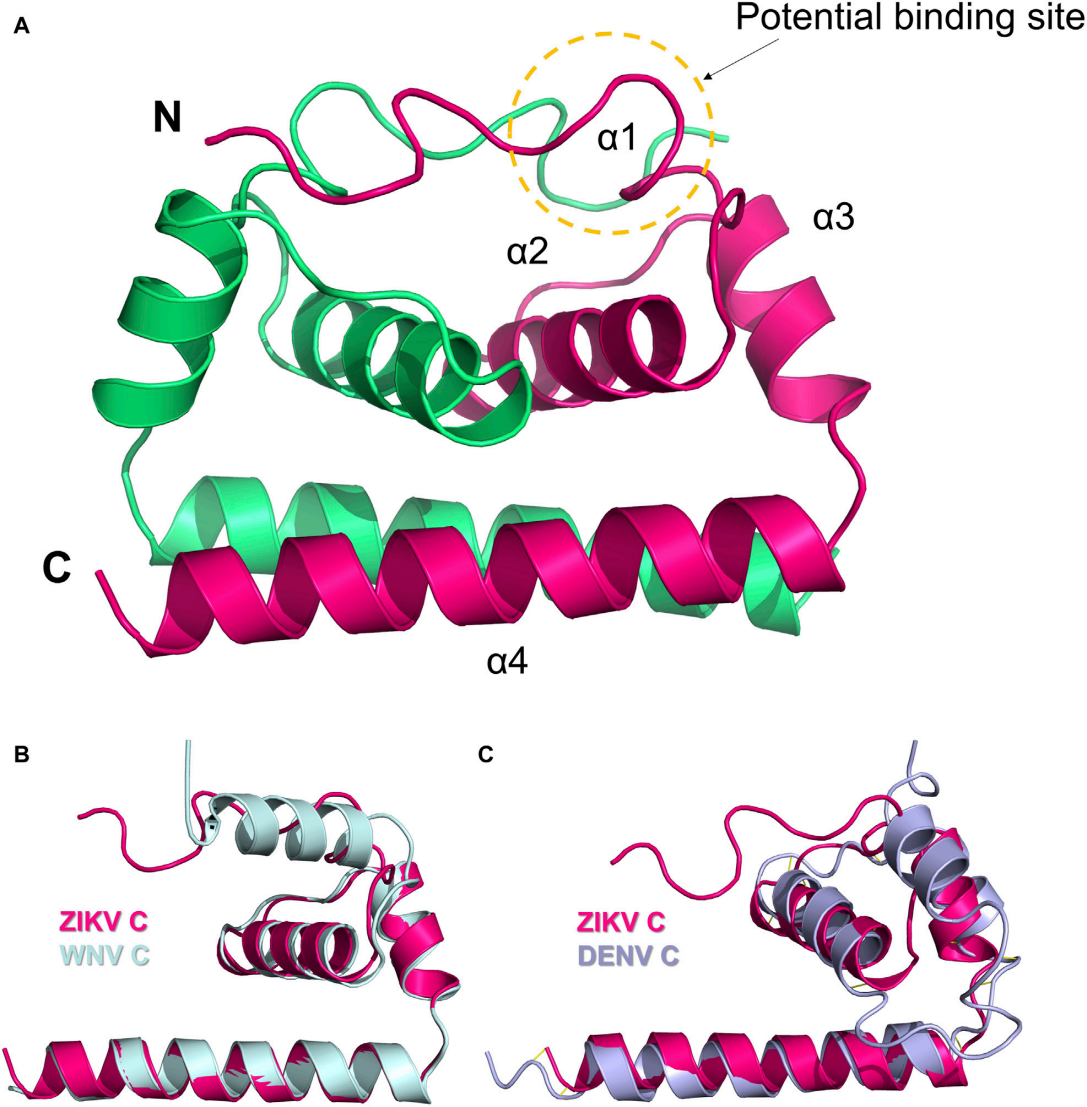
**(A)** Dimer structure of the C protein of ZIKV (5YGH). The two monomers are colored magenta and green. **(B)** Structural alignment of ZIKV C protein (magenta) and WNV C protein (light blue). **(C)** Structural alignment of ZIKV C protein (magenta) and DENV C protein (purple). Potential binding sites for inhibitors are indicated.

The C protein of ZIKV comprises four α-helices and a lengthy N-terminal loop, with each of the 2 C proteins combining to create a dimer (Figure 5A) (Shang et al., 2018). The distinctive N-terminal loop of the C protein enhances the strong bonding of the dimer assembly and forms a specific hydrophobic barrier at the lipid bilayer interface. The hydrophobic layer is believed to be the site of interaction with both the prM protein and the E protein. Hydrogen bonding at the N-terminal loops of the dimer helps to maintain the dimer’s shape. The N-terminal loop in the C protein is distinct from those found in the structures of WNV and DENV C. The interface, composed of two α4 helices, possesses a significant positive charge. It is believed that this interface is crucial for the binding of nucleotides, including either RNA or DNA. Apart from the N-terminal loop, the α2 ∼ α4 helices of the ZIKV C protein show a high similarity to those of WNV and DENV. The structure of ZIKV C is more similar to that of WNV C than DENV C, in line with the protein sequence similarity profile (47.2% vs 39.8%) (Shang et al., 2018).

Currently, no therapeutic targets or inhibitors have been identified for the C protein of ZIKV, but the potential to target C proteins has been confirmed in various viruses such as HBV, HIV, and other flaviviruses like DENV (Oliveira et al., 2017). The C protein of ZIKV contains a distinctive N-terminal loop. Mutations in the hydrophobic interfacial residues could change the binding properties of ZIKV to biofilm and lipid droplet structures (Shang et al., 2018). Hence, when researchers develop drugs that target the ZIKV C protein, the N-terminal loop should be regarded as a potential target, along with developing targets shared with typical flaviviruses (Figures 5B, C).

### 3.2 Non-structural proteins

In addition to the three above-mentioned structural proteins, ZIKV viruses also include seven nonstructural proteins: NS1, NS2A, NS2B, NS3, NS4A, NS4B, and NS5. NS1 is a virulence factor that serves multiple tasks. NS3 plays a crucial role in peptide processing and genome replication by cleaving the virally translated polyprotein into different NS proteins. NS5 comprises an N-terminal methyltransferase (MTase) structural domain and a C-terminal RNA-dependent RNA polymerase (RdRP) structural domain. NS2A, NS4A, and NS4B play a role in human viral pathogenesis and immunological responses. Because the structures of NS2A, NS4A, and NS4B are yet unidentified, only the NS1, NS2B, NS3, and NS5 protein structures and targets are described in this paper.

#### 3.2.1 NS1

NS1 is involved in multiple stages of the ZIKV life cycle, such as viral RNA replication and vesicle formation. NS1 interacts with crucial host proteins involved in RNA processing, gene expression regulation, and cellular homeostasis, significantly affecting viral replication and immunological responses. Furthermore, NS1 can behave as an immune evasion factor, enabling viruses to evade host immune monitoring. NS1 can also exacerbate disease advancement by causing endothelial damage, vascular leakage, and immunological reactions. NS1 alters endothelial glycocalyx and intercellular connections in the host cell, leading to the development of inflammatory cytokines and host autoantibodies, which further results in further exacerbation of disease (Perera et al., 2024).

**FIGURE 6:**
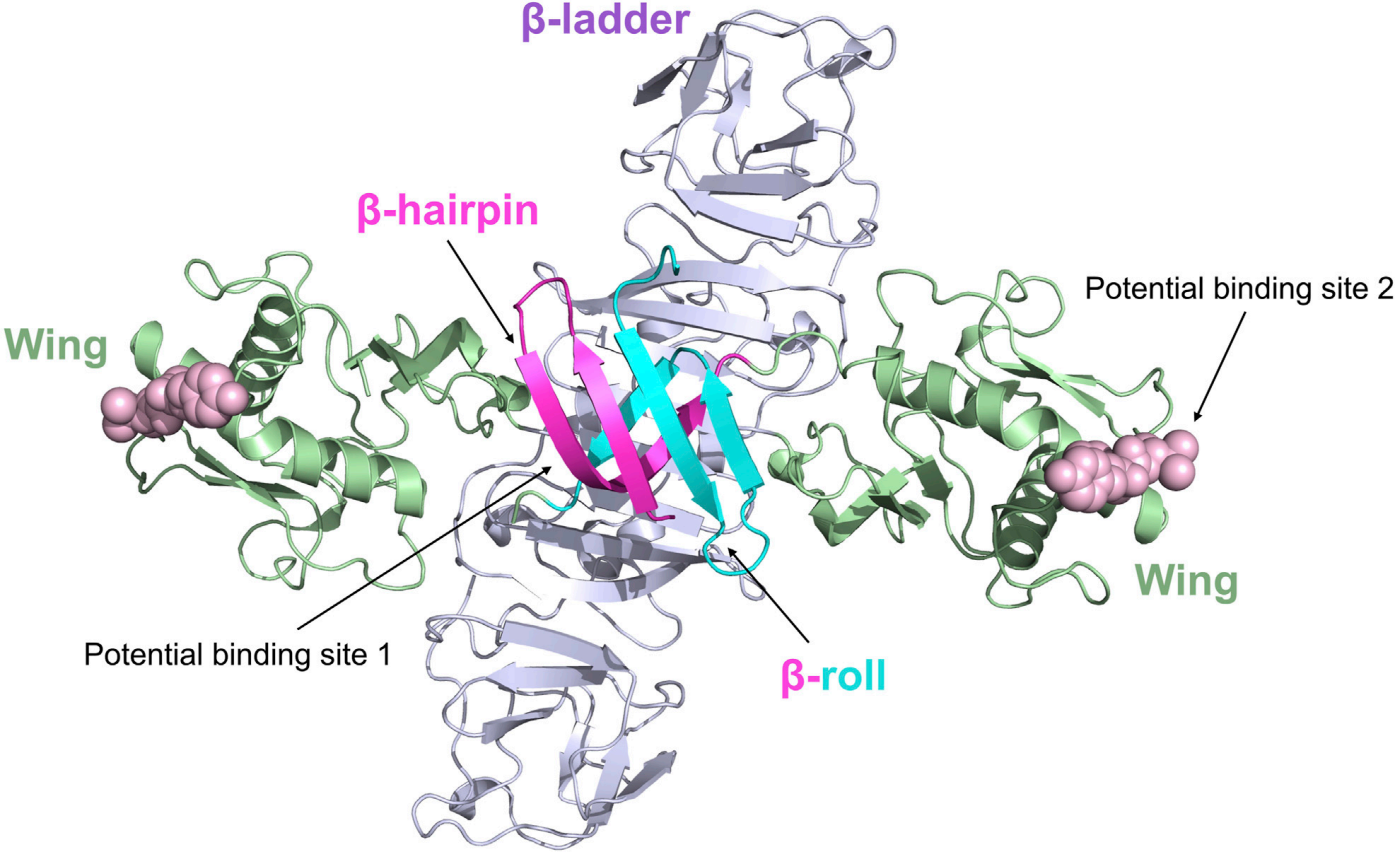
Ribbon representation of the ZIKV NS1 dimer (5GS6). The β-hairpin domains (residues 1–29) are shown in red and blue, respectively, for distinction; the Wing domain (residues 30–180) is shown in green; the β-ladder domain (residues 181–352) is shown in purple. Potential binding sites 1 and 2 of inhibitors are represented by spheres.

The crystal structure of NS1 in ZIKV is dimer and very similar to the NS1 structures of WNV and DENV2 (Figure 6) (Song et al., 2016). ZIKV NS1 consists of the following three structural domains in each monomer: the β-hairpin structural domain at the N-terminal, the Wing structural domain, and the β-ladder structural domain at the C-terminal (Prasad et al., 2017). In the dimer, the β-hairpin structural domain (amino acids 1–30) of two monomers bind together by their snap-like structures (including amino acids Tyr122, Phe123, and Val124) to create a rolled shape known as the “β-roll” dimerization domain (Xu et al., 2016).

There are no medications specifically designed to target ZIKV NS1, and the drug targets of ZIKV NS1 remain unclear. Recently, Raza et al. identified a potential action site (Potential binding site 1) inside the ZIKV NS1 protein by virtual docking (Raza et al., 2020). Their proposal suggests that the action site is situated in close proximity to the β-roll of the NS1 dimer, mostly consisting of residues Ser7, Asp9, Phe10, Ser11, Arg16, Thr19, and Val21. Since this study was conducted exclusively using computer simulation experiments, further validation is required to confirm the NS1 drug action site. Xu et al. discovered a complex loop in the wing domain of ZIKV NS1, containing a hydrophobic “spike” made up of Tyr122, Phe123, and Val124 (Xu et al., 2016). This structure may contribute to the binding of the virus to the host cell membrane. Flaviviruses have diverse amino acid sequences for the “spike,” but they all have a common trait of being hydrophobic or positively charged, which helps them adhere to membranes and could be a target for antiviral treatments (Potential Binding site 2).

#### 3.2.2 NS2B-NS3

The NS2B-NS3 protease is a chymotrypsin-like serine protease and is crucial in the ZIKV life cycle. The NS2B-NS3 protease aids the virus in proliferation by cleaving the viral polyprotein into structural and nonstructural proteins (Hilgenfeld et al., 2018). NS2B comprises three structural domains: an N-terminal, a C-terminal transmembrane helix, and a central hydrophilic region. The NS3 protease is a serine protease with protease and RNA helicase domains located at the N-terminal and C-terminal ends, respectively. NS2B and NS3 must be connected to perform well in their physiological functions (Leung et al., 2001).

**FIGURE 7:**
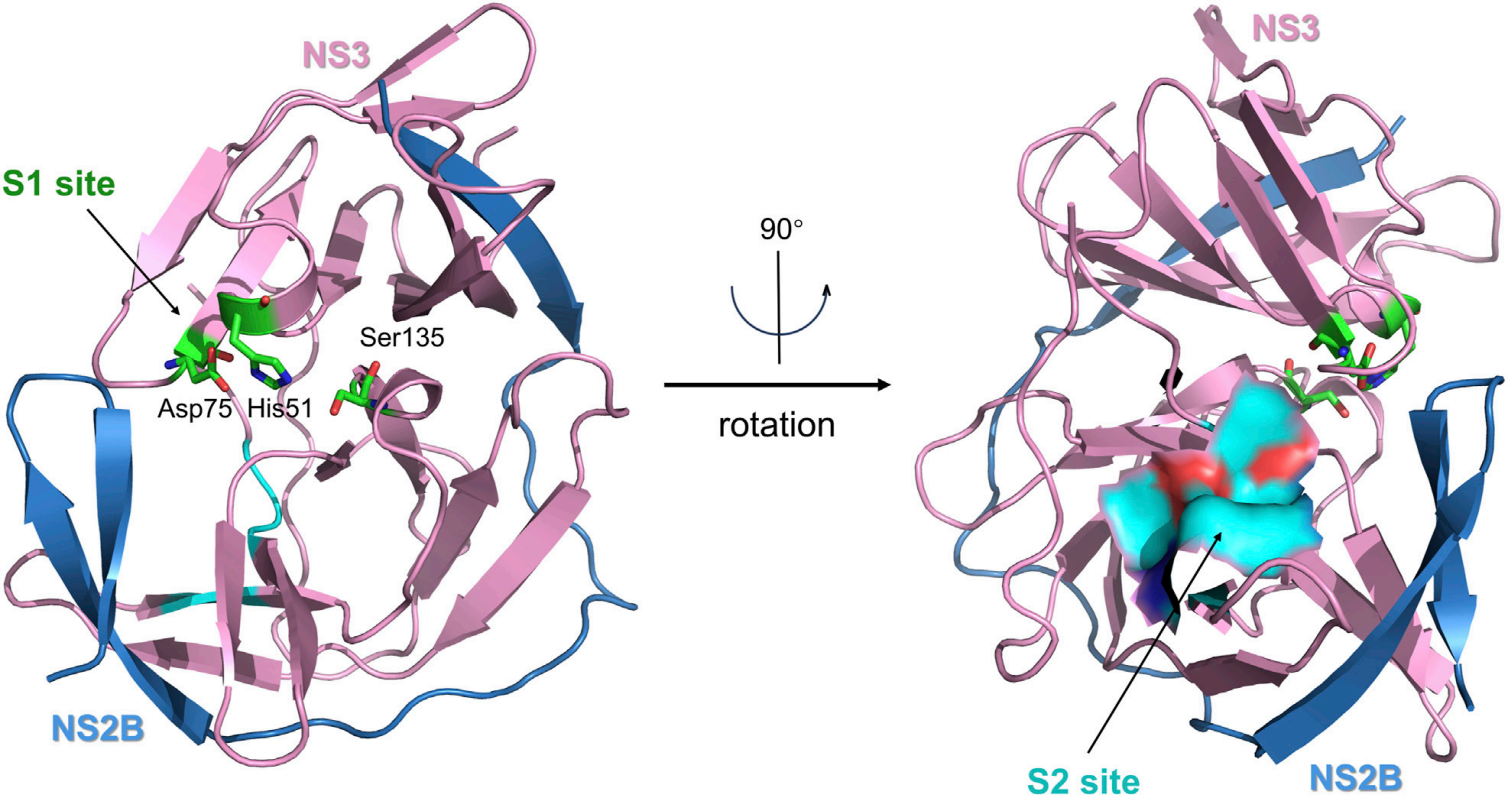
The X-ray crystal structure of the ZIKV virus protease NS2B-NS3 in its catalytically active conformation (PDB ID: 5LC0, ribbon diagram). NS2B is colored blue, and NS3 is pink. The S1 site (catalytic triad) is highlighted in green, and the S2 site (allosteric pocket) is highlighted in blue.

When not bound to a ligand, the ZIKV NS2B-NS3 protease has an open conformation, and the C-terminal portion of NS2B is disordered (Chen et al., 2016). When exposed to a peptidomimetic boronic acid inhibitor (CN-716), the ZIKV NS3 protease transitions to a closed conformation. NS2B then envelops NS3, creating a substrate-binding pocket that interacts directly with the inhibitor (Kuiper et al., 2017; Pant et al., 2023). The substrate-binding pocket of the ZIKV NS2B-NS3 protease contains two key catalytic sites—S1 and S2 (Figure 7). Lei et al. showed that the S1 site in the NS2B-NS3 protease is situated within the cavity at the center of NS3, which contains a catalytic triad composed of Ser135, His51, and Asp75 (Lei et al., 2016). The catalytic triad is hydrophilic, which creates robust hydrogen bonding connections with the ligand (Nitsche et al., 2014). The S2 site is located within NS2B and is mainly composed of the amino acids Ile123, Gly124, Ala164, Ile165, and Thr166 (Kang et al., 2017). Peptide mimetics or peptide-based covalent inhibitors mainly target the S1 site of the NS3 protein, with only a small portion interacting with S2 through intermolecular forces (Nitsche, 2019). This discovery offers a fresh possibility for the development of NS2B-NS3 inhibitors by introducing new targets.

Nowadays, various inhibitors targeting ZIKV NS2B-NS3 have been reported, such as peptide-mimetic or peptide-based covalent inhibitors, repurposed medicines, small-molecule inhibitors, and natural products. The NS2B-NS3 protease is recognized as a prominent therapeutic target among ZIKV treatments (Xiong et al., 2022).

#### 3.2.3 NS5

The NS5 protein is the largest and most conserved protein in ZIKV, with a molecular weight of around 100 kDa. It is involved in three crucial functions during the ZIKV life cycle: genome replication, capping, and interferon inhibition. New research indicates that it may also be involved in controlling the activity of the spliceosome (Wang et al., 2018). The protein structure of NS5 comprises the MTase structural domain at the N-terminus and the RdRP structural domain at the C-terminus.

The NS5 MTase of ZIKV is a dual-substrate enzyme that selectively binds to both RNA and S-adenosylmethionine (SAM). The flavivirus RNA is an 11-kB positive-sense, single-stranded RNA genome with a methylated 5’cap structure (N7MeGpppA2’OMe; Me, methyl group). This RNA is functionally stable, translationally efficient, and can evade the host immune response. The main function of MTase is to perform the methylation process at the N7 and 2′O sites of this RNA. On one hand, NS5-MTase methylates the N7 atom of guanosine and subsequently methylates the 2’O atom of the initiating adenosine in the nascent viral transcript (GpppA-RNA→N7MeGpppA-RNA→N7MeGpppA2’OMe-RNA). Mutations in MTase have been shown to cause methylation abnormalities in the N7 atom, resulting in flavivirus mortality. On the other hand, deficiencies in 2’O methylation also result in reduced a viral ability to survive (Jain et al., 2017). Moreover, MTase can use guanosine triphosphate (GTP) as a substrate to create a covalent NS5-GMP (guanosine diphosphate) intermediate, which subsequently transfers GMP from NS5 to the end of the receptor RNA transcript. These characteristics are essential for the survival of ZIKV and make MTase a desirable target.

**FIGURE 8:**
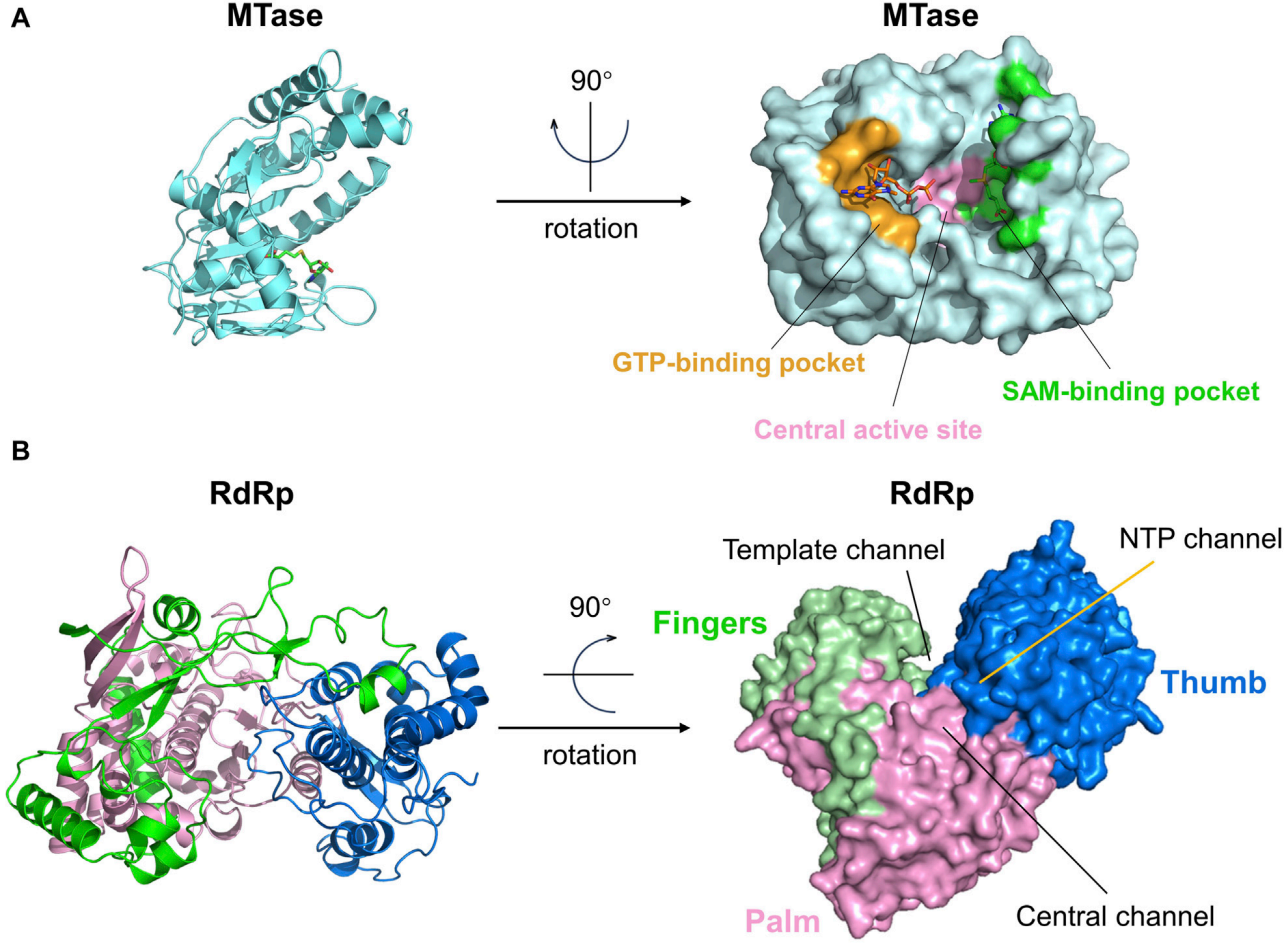
**(A)** The structure and active sites of the ZIKV Mtase domain. The central active site, GTP-binding pocket, and SAM-binding pocket are respectively represented in pink, yellow, and green. GTP and SAH molecules are bound to the GTP-binding pocket and SAM-binding pocket, respectively. **(B)** Structure and active sites of the ZIKV RdRp domain. The palm, fingers, and thumb regions of the RdRp domain are denoted in pink, green, and dark blue, respectively. The Central channel, Template channel, and NTP channel are as marked in the figure.

The MTase contains three distinct active sites: the central active site, the GTP-binding pocket, and the SAM-binding pocket (Figure 8A). Amino acids near the center active site are Lys61, Lys182, Asp146, and Glu218. GTP and SAM (SAH) are two types of ligands that frequently appear in the reported crystal structures of NS5 Mtase proteins (Zhao et al., 2017). The GTP-binding site is located in the N-terminal semi-open structural domain, which is closely associated with the guanylate transferase (GTase) activity of NS5 (Boonyasuppayakorn and Padmanabhan, 2014). Important amino acids involved in this part of the structure include Lys13, Asn17, Phe24, Lys28, and Ser152. The SAM-binding site is located in the C-terminal closed structural domain, and important amino acids include Asp79-Trp87, His110, and Asp131. SAM acts as a methyl donor in flaviviruses, transferring methyl groups to substrates when it binds to MTase. Eventually, SAM transforms into SAH (Zhang et al., 2017). Duan et al. found that SAM site binding is more stable and inhibition is more effective (Duan et al., 2017). Furthermore, even when the GTP site is occupied by a ligand, the SAM site can still bind the ligand, which makes it more attractive to develop inhibitors targeting the SAM binding site. All three active pockets in MTase have the potential to serve as sites for drug binding. However, only the GTP-binding pocket and the SAM-binding pocket have been reported to have ligands. Therefore, it is easier to obtain potential inhibitors of the NS5 MTase protein from these two binding pockets (Wang et al., 2018).

The RdRP, located on ZIKV NS5, is a key enzyme responsible for viral RNA transcription and replication (Figure 8B). The RdRP utilizes the viral genomic RNA as a template and synthesizes a complementary new RNA strand based on the sequence of the template. Within host cells infected by ZIKV, RdRP transcribes viral genomic RNA into messenger RNAs (mRNA), which are then used by the host cell to produce viral proteins. These procedures are essential for the viral life cycle.

Like other viral RdRP, the protein structure of flavivirus NS5 RdRP (PDB ID: 5WZ3) can be divided into three domains: the thumb, palm, and finger substructural domains (Figure 8B). These three parts form three channels on the protein surface: 1. “Central channel” for guiding the template and newly synthesized RNA, 2. “Template channel” for template RNA binding, and 3. “NTP channel” for NTP entry. These three channels work together to enhance nucleotide synthesis (Zhao et al., 2017). Currently, only the NTP channel has been identified as a target for drugs against the ZIKV NS5 RdRp protein, whereas the drug binding sites on the other two channels have not yet been explored (Upadhyay et al., 2017; Gharbi-Ayachi et al., 2020). The NTP channel contains key residues such as Arg739, Thr796, and Thr768, and inhibitors primarily function by forming hydrogen bonds with these residues (Gharbi-Ayachi et al., 2020).

Comparison of the NS5 protein structures of ZIKV, JEV, and ENV2 showed that the MTase and RdRp structural domains are quite similar in all three, differing only in the relative positions of these two domains. Thus, NS5 inhibitors that target JEV and ENV2 are frequently considered as viable medications for inhibiting ZIKV NS5. Exploring the usage of RdRP inhibitors designed for other flaviviruses on ZIKV is a crucial focus for discovering NS5 RdRP inhibitors (Zhao et al., 2017).

## 4 ZIKV drugs

Researchers have analyzed various potential targets in the ZIKV after conducting pharmacological experiments for years. Currently, the protein structure and function of ZIKV are poorly understood, so drug development for ZIKV has focused on E proteins, NS2B-NS3 proteins, and NS5 proteins. No drugs against ZIKV have been approved for marketing until now; however, various broad-spectrum and targeted drugs against the virus or host cells are currently under investigation. We provide an overview of the effective ZIKV inhibitors published in recent years and classify them according to their targets of action and structural sources. This work primarily documents IC_50_ and K_i_ data for protease inhibition and EC_50_ and CC_50_ data for cell activity (Gorshkov et al., 2019).

### 4.1 Protein inhibitors

**FIGURE 9:**
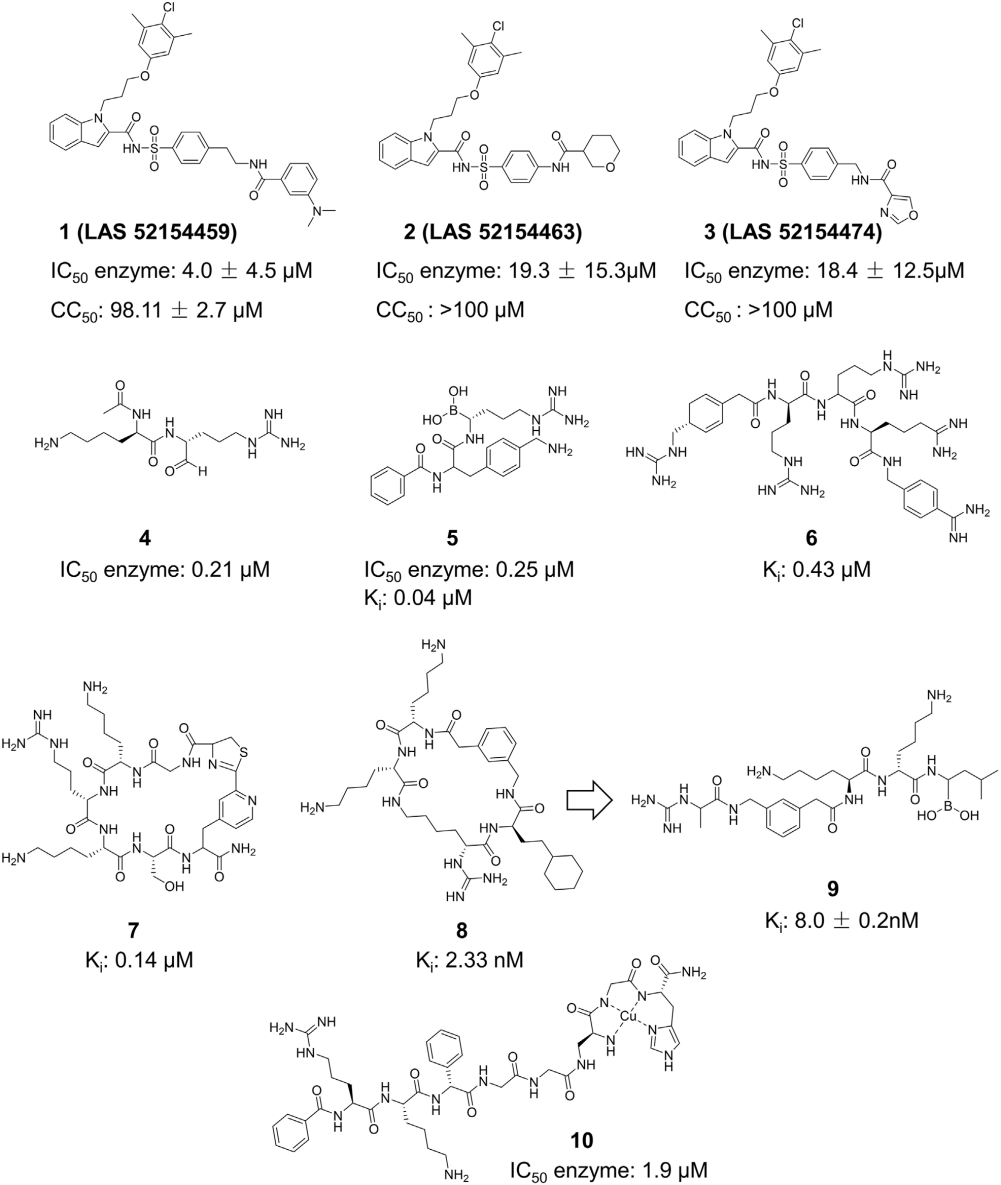
Chemical structures and bioactivities of Compound **1**–**10**.

The active site of the E protein is currently not clearly defined, and its inhibitors are primarily discovered by high-throughput screening (HTS) and have been infrequently published (Figure 9). Pitts et al. performed HTS on commercially available libraries of nucleoside analogs using the AlphaScreen methodology (Pitts et al., 2019). They identified seven more effective inhibitors of the ZIKV E protein, with IC_50_ values between 0.9 and 19.3 μM. Considering the activity and toxicity comprehensively, the most promising compounds were Compound **1** (LAS 52154459), Compound **2** (LAS 52154463), and Compound **3** (LAS 52154474). Their inhibitory capacity (IC_50_) for the E protein was 4.0 ± 4.5, 19.3 ± 15.3, and 18.4 ± 12.5 μM, respectively. These compounds inhibit the membrane fusion process mediated by E proteins and enhance the attachment of E protein to tryptic protease in the host cell’s cytoplasm. This results in hydrolytic digestion of viral capsid proteins, therefore blocking the fusion between viral and host endosomal membranes.

### 4.2 NS2B-NS3 protease inhibitors

The NS2B-NS3 protease of ZIKV plays a vital role in viral development and reproduction, making it a highly researched focus (Kang et al., 2017). NS2B-NS3 inhibitors are primarily categorized into four primary groups: peptide derivatives, repurposed pharmaceuticals, synthetic small molecules, and natural products. This paper categorizes existing ZIKV NS2B-NS3 protease inhibitors based on their site of action: competitive inhibitors (binding to the internal active site of NS2B-NS3) and noncompetitive inhibitors (binding to external active sites) (Voss and Nitsche, 2020).

#### 4.2.1 Competitive inhibitors (binding to the internal active site of NS2B-NS3)

##### 4.2.1.1 Peptide derivatives

Peptide inhibitors targeting ZIKV NS2B-NS3 are mainly generated from the protease’s substrate. All peptide derivative inhibitors mentioned include a minimum of two basic amino acid side chains (arginine, lysine, or analogs), which guarantees their accurate recognition of the S1 site of ZIKV NS2B-NS3 (Figure 9) (Shiryaev et al., 2023). Li et al. described Compound **4**, which features a characteristic arginine and lysine structure (Li et al., 2017). X-ray crystallography (PDB ID: 5H6V) verified the creation of a covalent bond between this compound and Ser135 of ZIKV NS2B-NS3, resulting in enhanced inhibitory activity (IC_50_ = 0.21 µM). Nitsche et al. created a new class of dipeptide structures by including a boronic acid structure to produce reversible covalent dipeptide boronic acid compounds (Compound **5**) (Lei et al., 2016). Compound **5** had improved anti-ZIKV activity with an IC_50_ of 0.25 µM and a K_i_ of 0.043 µM. Moreover, Compound **5** exhibited remarkable activity owing to the formation of a covalent bond between the boronic acid group and Ser135 of ZIKV NS2B-NS3. Compound **6** is a peptidomimetic molecule created by Phoo et al. (Phoo et al., 2018). The compound has four peptide fragments and includes two benzamide structural fragments, illustrating the interaction between the longer peptidomimetic inhibitor and NS2B-NS3. Compound **7** is the initial macrocyclic peptidomimetic molecule known to attach to the S1 site, which includes many arginine and lysine residues (Nitsche et al., 2019). The cyclic structure enhanced the stability of the peptidomimetic molecule (K_i_ = 0.14 µM), and extended its hydrolysis time from 1 to 20 h. Compound **8** is a cyclic peptidomimetic molecule described by Braun et al., in 2022 (Huber et al., 2022). It features a cyclohexylalanine residue with arginine and lysine. Compound **8** exhibited high activity with an affinity (K_i_ value) of 2.33 nM for NS2B-NS3. In 2023, Braun et al. created a non-cyclic peptidomimetic peptide inhibitor (Compound **9**) by modifying Compound **8** through breaking the cyclic structure and incorporating a boron structure (Braun et al., 2023). The chemical exhibited a K_i_ value of 8.0 ± 0.2 nM and formed a covalent bond with the ZIKV NS2B-NS3 linkage in a covalent binding manner. Compound **10** is a metallopeptide that binds to the catalytic triad. It fused the recognition structure on NS2B-NS3 by copper ions, leading to irreversible inactivation of NS2B-NS3 due to oxidative damage. Compound **10** shows potent inhibition against ZIKV NS2B-NS3 serine protease with an IC_50_ of 1.9 μM and has a high affinity for the target with a KD of 2.0 μM, making it a good candidate for further research and development (Pinkham et al., 2018).

##### 4.2.1.2 Repurposing drugs

Several drugs previously used for different diseases have also been tested for their anti-ZIKV activity, with some demonstrating superior effectiveness. The compounds listed in Table 1 are mainly antimicrobials and anti-HCV drugs.

**TABLE 1:** Chemical structures and bioactivities of Compound **11**–**17**.

No.	Compounds	Structures	Anti-ZlKV activity (µM)	Cytotoxic activity Cell line	CC_50_ (μM)	Classification	References
**11**	Bromocriptine	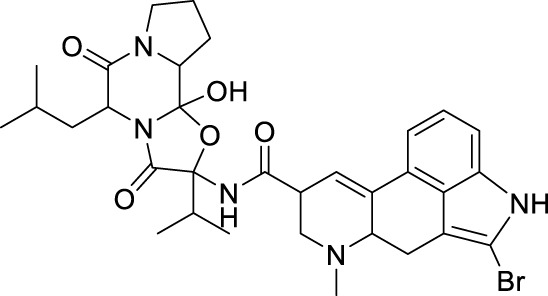	EC_50_: 13.04 ± 2.00	Vero	>40	Dopamine agonists	Shiryaev et al. (2017)
**12**	Novobiocin	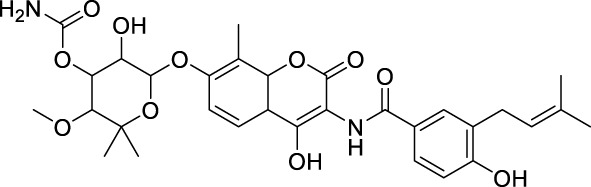	EC_50_: 42.63	Vero	1,388.02	Antibacterial agent	Yuan et al. (2017)
**13**	Montelukast Sodium	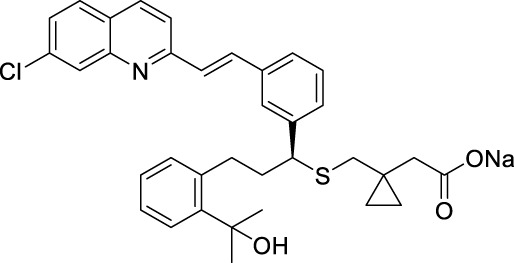	IC_50_ enzyme: 1.35 ± 0.17	Vero	46.22 ± 3.25	Antiallergic agent	Chen et al. (2019)
**14**	Pedalitin	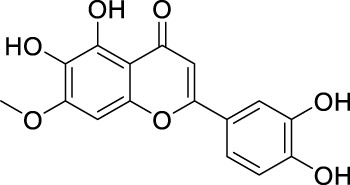	EC_50_: 52	Hyh-7	113.6	α-Glucosidase inhibitors	Chen et al. (2019)
**15**	Asunaprevir	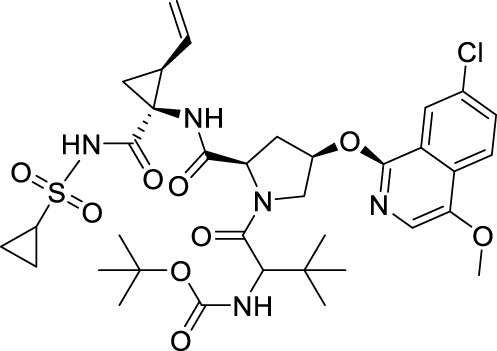	EC_50_: 4.7	Vero	30	Anti-HCV agent	Civitico et al. (1990)
**16**	Simeprevir	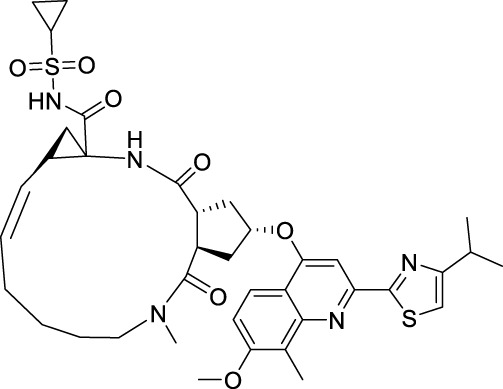	EC_50_: 0.4	U-87	10.1	Anti-HCV agent	Civitico et al. (1990)
**17**	Doxycycline	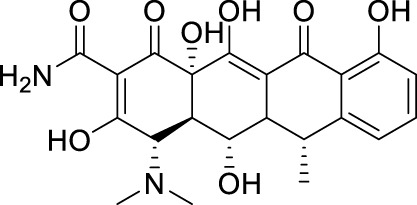	IC_50_: 5.3	—	—	Antibacterial agent	Chong Teoh et al. (2021)

##### 4.2.1.3 Synthetic small molecules

**FIGURE 10:**
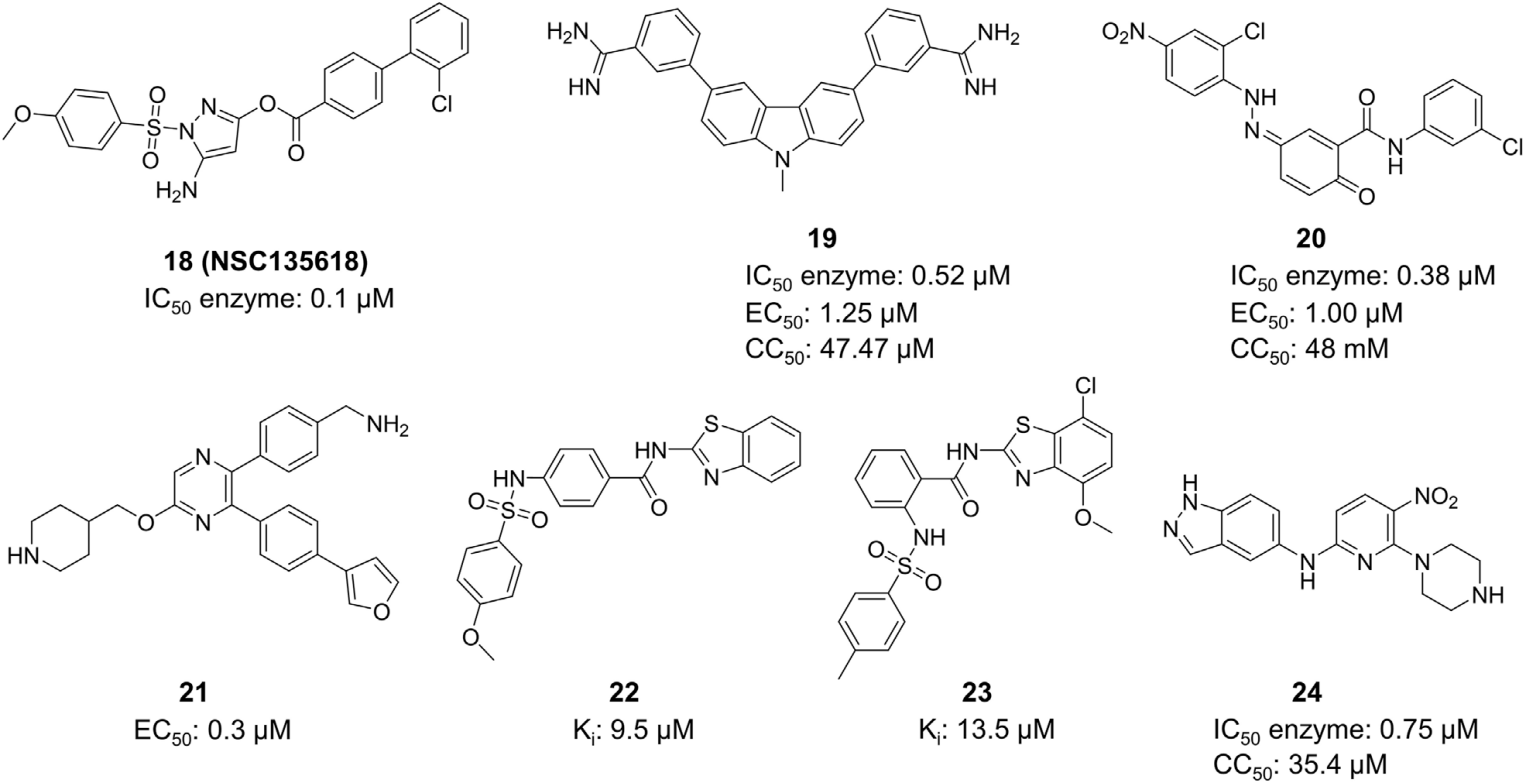
Chemical structures and bioactivities of Compound **18**–**24**.

Li et al. created Compound **18** (NSC135618), which stabilized the closed conformation of ZIKV NS2B-NS3 protease (bZiPro) by binding to it (Figure 10) (Zhang et al., 2016). Following the *in situ* hydrolysis of the ester link within Compound **18**, the benzoyl group creates a covalent connection with the catalytic residue Ser135. The other fragment does not exhibit any notable molecular interaction with NS2B-NS3 protease. The K_i_ value of Compound **18** for inhibiting the protease was 36.35 ± 25.7 µM. This study elucidates the precise mechanism of a covalent inhibitor, offering valuable insights for the advancement of ZIKV protease inhibitors. This compound has been utilized as a standard in numerous ZIKV medication investigations owing to its exceptional activity. Rassias et al. conducted a skeletal transition based on Compound **18** and discovered that specific carbazole derivatives with amidine groups exhibited notable anti-ZIKV activity, resulting in the novel N-substituted carbazolyl amidine Compound **19** (IC_50_ = 0.52 mM, EC_50_ = 1.25 mM, CC_50_ = 47.47 mM) (Rassias et al., 2019) Compound **20** (NSC135618) is a potent flavivirus protease inhibitor identified by virtual screening (Brecher et al., 2017). It effectively decreased the ZIKV titer in A549 cells *in vivo* with IC_50_ = 0.38 mM, EC_50_ = 1.00 mM, and CC_50_ = 48 mM.

X-ray crystallographic studies revealed that Compound **21** (SYC-1307) attaches to a flexible hydrophobic pocket in the ZIKV NS3 protein and maintains the protease in an open, catalytically inactive state (Figure 10) (Yao et al., 2019). This compound exhibits an IC_50_ value of 0.2 µM against the ZIKV protease and an EC_50_ value of 0.3 µM against ZIKV FLR strain-infected U87 cells. Lee et al. conducted virtual screening on 40,967 compounds to choose 71 candidates, and identified HCV NS3/NS4A inhibitors from this group (Lee et al., 2017). Multiple compounds having inhibitory activity (IC_50_ around 5–10 µM) and binding affinity (KD) for ZIKV NS2B-NS3 protease (substrate Boc-Gly-Arg-Arg-AMC) were discovered. The competitive inhibitor Compound **22** and Compound **23** were determined to be the most effective molecule with a K_i_ value of 9.5 µM and 13.5 µM, respectively. Compound **22** and Compound **23** exhibit numerous similarities to Compound **18**, and the docking analysis revealed a distinct hydrogen bonding interaction between its benzene sulfonamide group and Ser135. Shin et al. discovered 123 candidates out of 4,67,000 compounds using virtual screening with the structure of ZIKV NS2B-NS3 protease (Shin et al., 2021). The most potent compound (Compound **24**) from the 123 potential inhibitors was subsequently screened. Compound **24** suppressed the replication of ZIKV (strain: PRVABC59) RNA in HEK-293 cells in a dose-dependent manner, exhibiting an IC_50_ value of 0.75 µM.

##### 4.2.1.4 Natural products

In past studies, numerous investigations have focused on screening natural products, revealing that a significant amount of flavonoids have anti-ZIKV activity. Lim et al. analyzed 22 polyphenolic compounds using recombinantly expressed NS2B-NS3 enzymes (Lim et al., 2017). They screened Compound **25** (Myricetin), **26** (Luteolin), **27** (Epicatechin gallate, ECG), **28** (Astragalin), **29** (Rutin), **30** (Gallocatechin gallate, CG), **31** (Epigallocatechin gallate, EGCG) and **32** (ZP10) for their inhibition to NS2B-NS3 protease *in vitro* using the Förster resonance energy transfer assay (FRET) (Table 2).

**TABLE 2:** Chemical structures and bioactivities of Compound **25**–**32**.

No.	Compound	Structure	Anti-ZlKV activity (µM)	Compound source	References
**25**	Myricetin	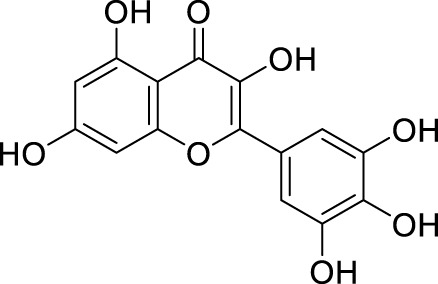	IC_50_ enzyme: 22 ± 0.2	Polyphenols	Lim et al. (2017)
**26**	Luteolin	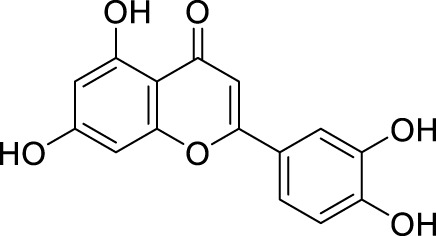	IC_50_ enzyme: 53 ± 1.3	Flavonoid	Lim et al. (2017)
**27**	Epicatechin gallate, ECG	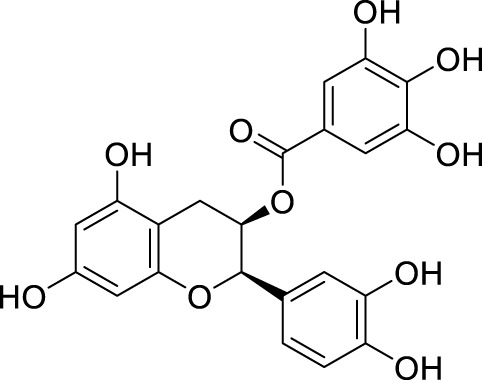	IC_50_: 89 ± 1.6	Tea and cocoa extract	Lim et al. (2017)
**28**	Astragalin	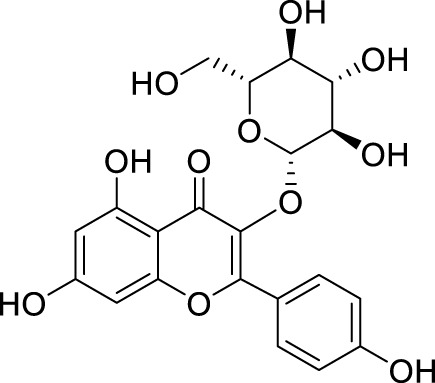	IC_50_ enzyme: 112 ± 5.5	Scutellaria L	Lim et al. (2017)
**29**	Rutin	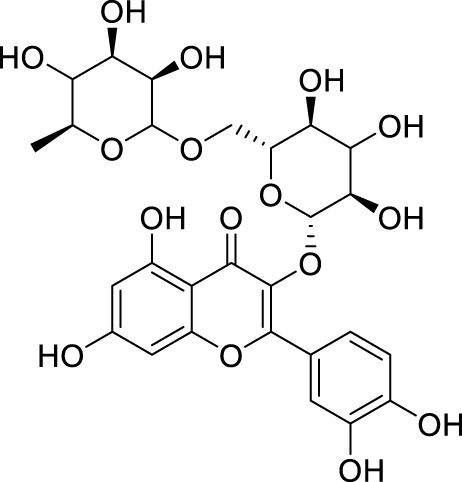	IC_50_ enzyme: 104 ± 2.9	Flavonoid	Lim et al. (2017)
**30**	Gallocatechin gallate, CG	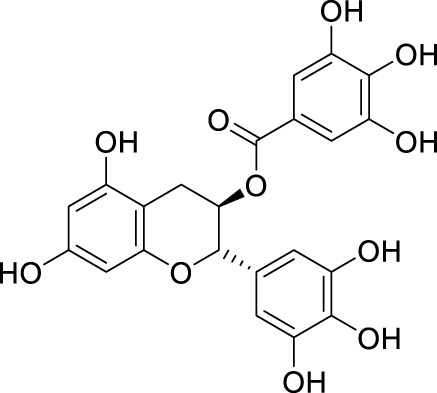	IC_50_ enzyme: 99 ± 1.8	Polyphenols, tea extract	Lim et al. (2017)
**31**	Epigallocatechin gallate, EGCG	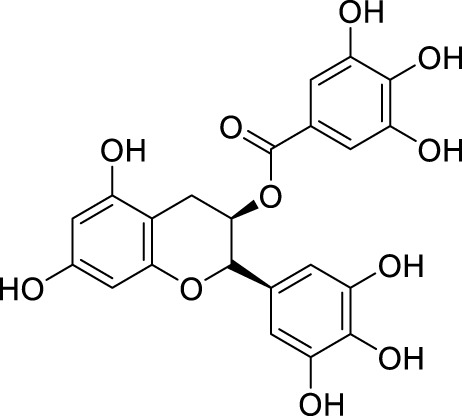	IC_50_ enzyme: 87 ± 1.2	Polyphenols, Tea extract	Lim et al. (2017)
**32**	ZP10 (theaflavin-3,3′-digallate)	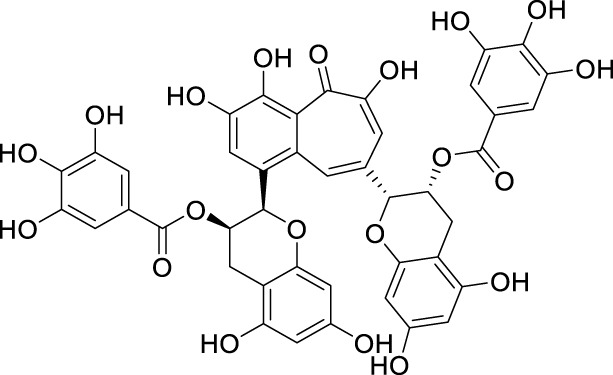	EC_50_: 7.65	Tea extract	Perachon et al. (1999)

#### 4.2.2 Noncompetitive inhibitors (binding to external active sites)

**FIGURE 11:**
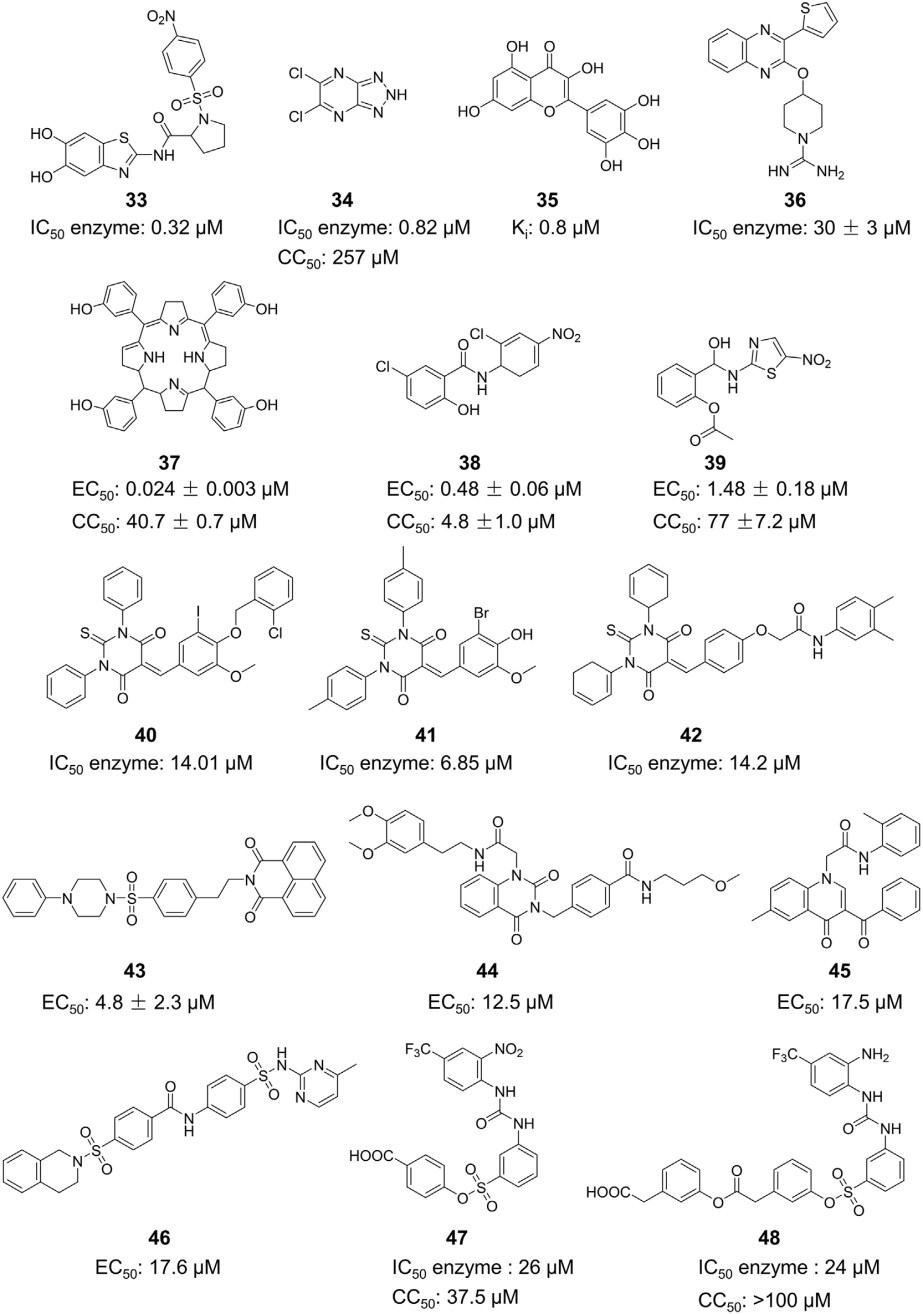
Chemical structures and bioactivities of Compound **33**–**48**.

Compound **33**, a proline-based variant inhibitor of NS2B-NS3 protease, showed potent inhibition of ZIKV NS2B-NS3 at a substrate concentration of 100 μM, with an IC_50_ value of 0.32 μM for the NS2B-NS3 enzyme (Figure 11) (Millies et al., 2019). Compound **34** was acquired by Shiryae et al. after screening a library of inhibitors targeting the WNV protease exosomes (Shiryaev et al., 2017). The IC_50_ value of Compound **34** is 0.82 μM when tested with a Pyr-RTKR-AMC substrate concentration of 100 μM. Flavonoids Compound **35** hinders ZIKV NS2B-NS3 in a noncompetitive manner. Simulation studies discovered that it attaches to the pocket located at the rear of the active site, however, this binding has not been verified by experiments (Roy et al., 2017). Most small-molecule modification inhibitors attach to a region near the active site to stabilize the conformation of NS3. This prevents NS2B-NS3 from closing state and keeps the protease in an open state. This mode of action offers new prospects for targeting the open conformation of NS2B-NS3 (Mahawaththa et al., 2017). Zephyr and his colleagues discovered a set of compounds containing a quinoxaline core using a fragment-based drug discovery (FBDD) (Zephyr et al., 2023). These compounds were identified by alanine-scanning mutagenesis at potential inhibitor binding sites, with Compound **36** being representative. Compound **37** (Temoporfin), Compound **38** (Niclosamide), and Compound **39** (Nitazoxanide) are all marketed drugs that have demonstrated some ability to inhibit the ZIKV in laboratory experiments (Nunes et al., 2022). Lin et al. discovered three compounds (Compound **40**, Compound **41**, and Compound **42**) using virtual screening from a commercial compound library (Lin et al., 2023). They assessed the inhibitory action of these compounds on the NS2B-NS3 protease and identified their binding sites as variant sites through virtual docking. All three drugs showed more inhibitory efficacy against NS2B-NS3 protease compared to the control substance Temoporfin (IC_50_ = 18.77 μM).

### 4.3 NS5 protein inhibitors

NS5 consists of the MTase domain and the RdRp domain. Below, inhibitors of NS5 are described based on these two binding sites.

#### 4.3.1 MTase inhibitors

Since the structure of MTase has not been reported, Stephen et al. constructed the three-dimensional structure of NS5 MTase protein through homology modeling (Figure 11) (Stephen et al., 2016). They then screened a molecular library with 28,341 compounds and confirmed through Plaque Reduction Assay on ZIKV-infected Vero cells that four compounds showed inhibitory activity against ZIKV. The inhibitory activities (EC_50_ values) of these compounds were as follows: **43** (F3043-0013) - 4.8 μM, **44** (F0922-0796) - 12.5 μM, **45** (F1609-0442) - 17.5 μM, and **46** (F1750-0048) - 17.6 μM (Delgado-Maldonado et al., 2023). Hernandez et al. screened Compound **47** and **48** by the FBDD method based on the crystal structure of ZIKV Mtase (Hernandez et al., 2019). The IC_50_ values of the enzymatic inhibitory capacity of the two compounds against ZIKV MTase were 26 μM and 24 μM, respectively, and the CC_50_ values were 37.5 μM and >100 μM, respectively. These compounds have a simple structure and strong inhibitory effect against DENV. Further optimization may lead to the discovery of novel inhibitors.

**FIGURE 12:**
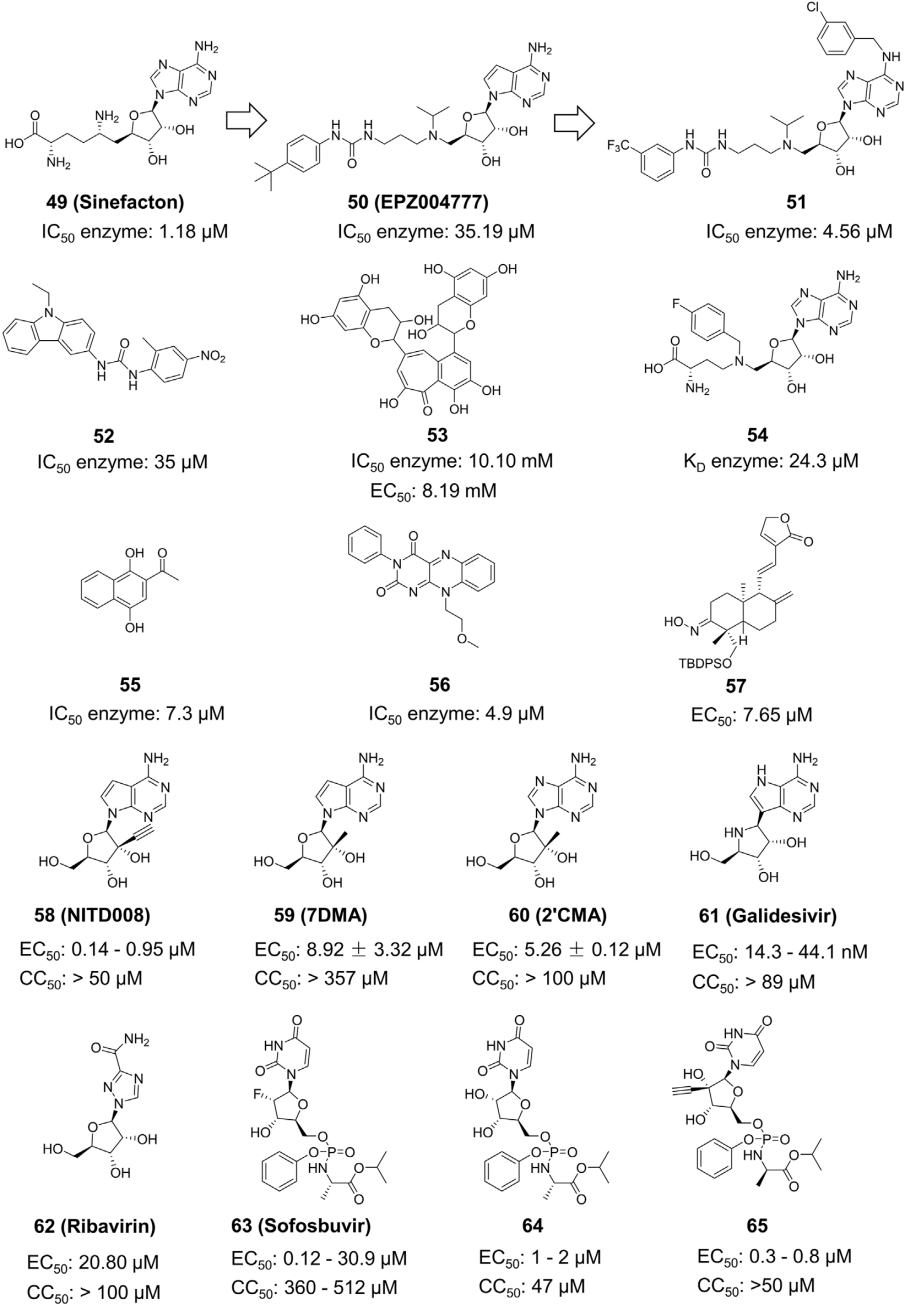
Chemical structures and bioactivities of Compound **49**–**65**.

Compound **49** (Sinefacton, SIN) is an antifungal antibiotic discovered by Eli Lilly and Company from the fermentation broth of Streptomyces griseus NRRL 3739. It also acts as an inhibitor of the ZIKV NS5 MTase with an IC_50_ value of 1.18 µM (Figure 12) (Coutard et al., 2017). It is structurally similar to SAM and acts as a competitive inhibitor of the MTase of several flaviviruses (WNV, DENV-2 and YFV). Hercik et al. confirmed the binding location of SIN by reporting the crystal structure of SIN bound to Mtase (Hercik et al., 2017). Tao and colleagues created a new SIN derivative, Compound **50**, based on SIN and Compound **50** (EPZ004777), which has a better anti-ZIKV effect (IC_50_ = 4.56 µM) compared to EPZ004777 (Tao et al., 2018). Although no mechanistic studies were performed on Compound **51**, it is expected have a similar mode of action as SIN.

Spizzichino and others developed the structure of ZIKV NS5 MTase through homology modeling. By using compounds from the NCI diversity database for molecular docking targeting the SAM pocket of NS5-MTase, they obtained a new set of carbazole urea inhibitors, among which Compound **52** had an IC_50_ of 35 µM (Figure 12) (Spizzichino et al., 2020).Compound **52** also shown a direct inhibitory impact on the DENV virus. Song et al. conducted High-Throughput Screening (HTS) on a natural product library targeting the SAM pocket (Song et al., 2021). They discovered that Compound **53** (Theaflavin) had a dose-dependent inhibitory upon ZIKV, with an IC_50_ value of 10.10 mM and an EC_50_ value of 8.19 mM. The SPR investigation indicated that Theaflavin exhibited a higher binding affinity towards wild-type MTase compared to the D146A mutant MTase, implying that D146 is the key amino acid in the interaction between MTase and Theaflavin. Jain et al. produced and isolated the ZIKV NS5 MTase with the LOBSTR (DE3) *Escherichia coli* strain. They then employed Isothermal Titration Calorimetry (ITC) to analyze and contrast the thermodynamic characteristics of SAM, SAH, and Compound **54** (MS 2042) when binding to NS5 MTase. MS2042 binds to ZIKV NS5-MTase with a KD value of 24.3 µM, while SAM and SAH had KD values of 2.58 µM and 2.87 µM, respectively. MS2042 demonstrates superior binding activity. Crystallographic analysis revealed that MS2042 reaches into RNA-binding channels typically filled by the 2′OH group of the RNA cap (Jain et al., 2017).

Samrat et al. created a HTS based on Fluorescence Polarization technology with a fluorescent counterpart of SAM (FL-NAH) (Samrat et al., 2023). Initial evaluation revealed two possible inhibitors of ZIKV NS5 MTase: Compound **55** (NSC111552) and Compound **56** (NSC288387). The binding research demonstrated that these two compounds had a strong affinity for directly binding to NS5 MTase. Furthermore, both drugs significantly decreased ZIKV replication in cellular tests at non-cytotoxic concentrations with IC_50_ values of 7.3 µM and 4.9 µM, respectively. These two compounds also shown superior inhibitory effects on DENV3. Qian et al. derivatized Andrographolide, a natural drug extract from Andrographis paniculata, to produce several 3-nitroso or 3-alcohol-19-indole dehydroepiandrosterone compounds (Qian et al., 2022). Plaque Reduction Assay was used to obtain a series of dehydroepiandrosterone derivatives, including Compound **57**, which inhibited the ZIKV NS5 MTase with an EC_50_ of 0.71 ± 0.72 µM and a CC_50_ more than 200 µM.

#### 4.3.2 RdRp inhibitors

The ZIKV RdRp protein’s structure was recently determined, and only a small number of inhibitors that target ZIKV RdRp have been identified. The majority of these inhibitors were discovered through screening existing RdRp inhibitors (Bernatchez et al., 2020). This review classifies these inhibitors into three classes according to their structural characteristics: nucleoside analogues, synthetic small molecule medicines, and natural products.

##### 4.3.2.1 Nucleoside analogs

Nucleoside analogs are the most classical RdRp inhibitors commonly utilized to treat various infections. Nucleoside analogs are converted to triphosphate form by kinase in the host, which subsequently fools the viral polymerase and is incorporated by the virus into its DNA or RNA. It then disrupts viral DNA or RNA synthesis and inhibits viral growth. Many nucleoside analogs effective against ZIKV are derived from chemicals previously proven useful against a variety of other viruses.

Deng et al. assessed the antiviral activity of Compound **58** (NITD008) against the ZIKV in Vero cells (Figure 12) (Deng et al., 2016). The Plaque Reduction Assay demonstrated that treatment with various concentrations of Compound **58** markedly decreased the amount of plaques. At a concentration of 5 mM, Compound **58** decreased the viral titers of two virus strains, GZ01/2016 and FSS13025/2010, by 400-fold and 1,000-fold, respectively. Compound **58** has EC_50_ values of 241 nM against GZ01/2016 and 137 nM against FSS13025/2010. Compound **58** was found to drastically decrease the death rate of A129 mice *in vivo* experiments. Nucleoside analog Compound **59** (7-deaza-2’-C-methyladenosine, 7DMA) had been investigated as a therapeutic compound for HCV and later failed in clinical trials. Zmurko et al. found that 7DMA has an inhibiting effect on ZIKV in Vero cells, with an EC_50_ value of 8.92 ± 3.32 µM (Zmurko et al., 2016). Compound **60** (2’-C-methyladenosine, 2’CMA) was first identified as an NS5 inhibitor for certain flaviviruses. However, it was later also discovered effectively inhibit ZIKV RdRp, with an EC_50_ value of 5.26 ± 0.12 µM (Eyer et al., 2016; Wang et al., 2018). Compound **61** (Galidesivir, also known as BCX4430) is a broad-spectrum inhibitor that can act against Ebola virus (EBOV), Marburg virus (MARV), and YFV. It effectively inhibits ZIKV *in vivo* and *in vitro* (Julander et al., 2017). Compound **61**’s wide-ranging activity makes it a promising drug candidate. Compound **62**, also known as Ribavirin, is a nucleoside analog commonly used to treat other viral infections including HCV. It has demonstrated anti-ZIKV effects in cellular and murine models of ZIKV infection with STAT1 gene deletion (Bernatchez et al., 2020).

Compound **63** (Sofosbuvir, a nucleotide analog inhibitor) appeared on the market in 2013 and is presently utilized in combination with ledipasvir (Harvoni^®^) to treat chronic HCV infection (Figure 12). Sofosbuvir provides efficient virus clearance, minimal side effects, and a shorter treatment duration compared to earlier HCV medications. Sofosbuvir inhibits ZIKV in Huh-7 cells with an EC_50_ value ranging from 1.37 μM to 4.6 μM, which is slightly lower than its inhibitory effect on HCV viruses with an EC_50_ of 0.5 μM–0.85 μM. In the future, Sofosbuvir may be utilized either independently or in combination for treating ZIKV (Ferreira et al., 2017). Compound **64** (2’-C-methyluridine aryoxyl phosphoramidate) and **65** (2’-C-ethynyluridine aryoxyl phosphoramidate) exhibit a structure analogous to sofosbuvir. Studies conducted *in vitro* have demonstrated that Compound **64** and **65** have higher anti-ZIKV effectiveness than Sofosbuvir (Bernatchez et al., 2019).

**FIGURE 13:**
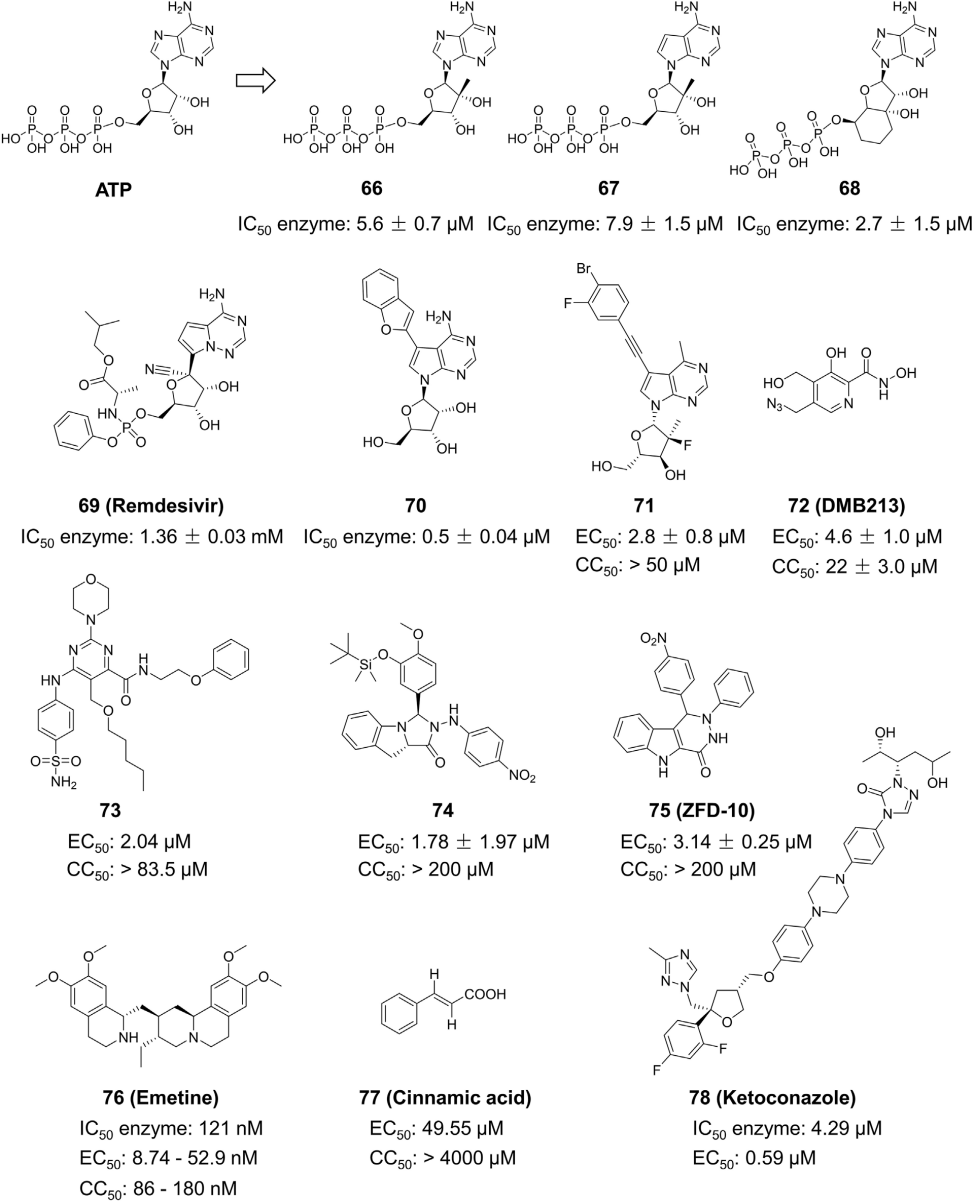
Chemical structures and bioactivities of Compound **66**–**78**.

Hercík et al. imitated the structure of ATP and created a series of nucleoside analogs featuring a triphosphate structure (Figure 13) (Hercík et al., 2017). In cell assays, the IC_50_ values of Compound **66**, **67** and **68** for anti-ZIKV reached 5.6, 7.9 and 2.7 μM, respectively. Compound **69**, also known as Remdesivir, is a wide-ranging antiviral medication initially created for treating Ebola virus. It was subsequently authorized for emergency use in treating COVID-19. Konkolova et al. investigated the inhibitory effect of Remdesivir targeting flavivirus RdRp, and its inhibitory capacity against ZIKV and DENV RdRp was shown to have an IC_50_ value of 1.36 ± 0.03 and 1.40 ± 0.14 mM, respectively (Konkolova et al., 2020). In 2021, Milisavljevic et al. produced a series of 7-deazaadenosine nucleoside analogs and tested their inhibitory effects on RdRp from various flaviviruses, such as ZIKV and DENV (Milisavljevic et al., 2021). One of the representative compounds exhibited *in vitro* inhibitory activity with IC_50_ values of 0.5 ± 0.04, 0.5 ± 0.06, and 0.3 ± 0.05 μM against ZIKV, JEV, and WNV RdRp, respectively. This investigation also showed that these compounds have enhanced inhibitory efficacy against ZIKV RdRp when formulated as triphosphates (Nascimento et al., 2021).

##### 4.3.2.2 Synthetic small molecules

Yao et al. developed a series of 6-methyl-7-ethynyl-7-deazaadenosine nucleoside analogs as novel inhibitors to ZIKV RdRp (Figure 13) (Yao et al., 2022). Compound **71** exhibited the most potent anti-ZIKV activity (EC_50_ = 2.8 ± 0.8 μM, CC_50_ > 50 μM) in an A549-based cell model, with an inhibitory activity approximately 5-fold higher than that of the positive control NITD008. Compound **71** demonstrated consistent inhibitory effects against various ZIKV strains in different cellular models including SNB19, A549, Huh7 and Vero. These results suggest its potential as a ZIKV therapeutic agent. Xu and his colleagues developed a novel method for determining RdRp using pure recombinant ZIKV NS5 polymerase (Bernatchez et al., 2020). Sofosbuvir and Compound **72** (DMB213) were tested for their anti-ZIKV activity using this approach, and their action site was determined to be ZIKV RdRp. The EC_50_ values for the two compounds were 8.3 μM and 4.6 mM, respectively (Xu et al., 2017). DMB213 showed superior activity compared to Sofosbuvir. DMB213’s cytotoxicity is suboptimal but offers promise for the development of subsequent compounds. DMB213 binds to RdRp in competition with natural nucleotides, inhibiting RNA synthesis. It has an IC_50_ value of 5.2 μM for inhibiting RdRp. The mutation of S604T in RdRp can deactivate soffebuvir, however it does not impact the function of DMB213. The anti ZIKV activity of Compound **73** is three times higher than that of nucleoside analogue 7DMA, and it has a good selectivity index (>41) (Bernatchez et al., 2020).

Zhou et al. studied the anti-ZIKV effects of tricyclic derivatives in indoline and imidazolidone (Zhou et al., 2023a). Compound **74** was discovered with an EC_50_ value of 1.78 ± 1.97 μM by Plaque Reduction Assay. This compound showed higher inhibition of the Asian ZIKV SZ-VIV01 strain compared to the African ZIKV MR766 strain. Zhou et al. identified a new small molecule Compound **75** (ZFD-10) in their recent study (Zhou et al., 2023b). This compound has a structure of 1H-Pyridazine [4,5-b] indole-4 (5H)-ketone, and the EC_50_ values are 3.71 ± 1.11 μM, 4.8 ± 0.11 μM, and 4.8 ± 0.9 μM to ZIKV-infected Vero, A549, and Huh7 cells, respectively.

##### 4.3.2.3 Natural products

Compound **76** (Emetine), is both a natural product and a commercially available medication used as a HER2 inhibitor (Figure 13). Yang et al. discovered that this drug reduced the activity of ZIKV NS5 RdRp directly with an IC_50_ value of 121 nM (Yang et al., 2018). Emetine significantly hindered viral replication in ZIKV-infected Vero cells with an EC_50_ of 8.74 nM. Chen et al. sought to utilize Compound **77** (Cinnamic acid) in anti-ZIKV research (Chen et al., 2021). They found that in ZIKV-infected Vero cells, it had an EC_50_ value of 49.55 μM and a CC_50_ value of 4,000 μM. Chen et al. conducted a virtual screening of anti-infective drugs using a natural product library to identify possible RdRp inhibitors (Chen et al., 2023). Further RdRp enzymatic testing, cytotoxicity assessment, and SPR testing determined that Compound **78** (Ketoconazole) effectively inhibited RdRp activity (IC_50_ = 4.29 µM) with an EC_50_ value of 0.59 µM in a Huh-7 cell model.

## 5 Summary and future directions

Because previous drug studies of ZIKV did not garner much research attention; therefore, no targeted drugs for ZIKV have been reported so far. However, since the large outbreak of ZIKV in 2016, the ZIKV has continued to spread on a large scale. Soon after, the World Health Organization declared the ZIKV outbreak a public health emergency of international concern. This has led to an urgent need for ZIKV-targeted drugs. This review outlines the life cycle, structure, and function of ZIKV, identifies the locations of therapeutic targets, and highlights representative anti-ZIKV drugs. Building on previous research, this paper consolidates the current state of studies on various ZIKV targets. This allows readers to gain a comprehensive understanding of the current state of research on ZIKV-related inhibitors and to quickly clarify the relationships between the structures of various inhibitors.

Over the past 5 years, there has been notable advancement in comprehending ZIKV because of the consecutive reporting of crystal structures of both nonstructural and structural ZIKV proteins. Compounds identified with potential activity have been mainly directed against ZIKV nonstructural proteins. This state of affairs is mainly because the non-structural protein of ZIKV is very similar to that of many other flaviviruses. Thus, a large number of compounds with inhibitory effects on flaviviruses such as WNV and DENV have also been shown to inhibit ZIKV. At this stage, drugs targeting the ZIKV mainly target two regions: NS2B-NS3 and NS5. Because NS2B-NS3 has unique substrates, most drugs designed against NS2B-NS3 are analogs of these substrates, and these drugs often contain structures of arginine and lysine. NS5 plays an important role in many flaviviruses, and many related inhibitors have been reported in other flaviviruses, so most of the drugs for ZIKV NS5 come from the reuse of other flavivirus drugs. No relevant inhibitors have been identified yet for NS1. It has been proposed that its active site might be situated in the Wing domain, making it a promising area for research.

In terms of structural proteins, other than a few scattered reports of inhibitors for E proteins, there have been no reports on prM and C protein inhibitors. This could be attributed to the prolonged absence of reported crystal structures of structural proteins. The specific locations where drugs act on the E, prM, and C proteins are still unknown, although several hypotheses have been proposed. As the prM protein has a natural ligand NAG, using the NAG as a lead compound for drug design may have a higher success rate. Researching inhibitors that target the ZIKV structural proteins is a promising direction for development.

The authors expect that continued research into NS2B-NS3 and NS5 inhibitors from current compounds will be the most effective method for developing ZIKV medications for the foreseeable future. Additionally, developing treatments with broad-spectrum activity against ZIKV and other viruses will be a more beneficial approach. Highly effective inhibitors for ZIKV NS2B-NS3 and ZIKV NS5 have been identified, with EC_50_ values < 1 µM. These inhibitors have potential for further improvement in the future. Peptide analogs representing NS2B inhibitors and nucleoside analogs representing NS5 inhibitors, which are generally more potent, are anticipated to continue being the primary focus of ZIKV medication research in the future.
